# Microplastics Contamination and Ecological Risk Assessment in Natural and Afforested Mangrove Stands of Bahrain: Abundance, Polymer Composition, and Onshore–Offshore Variability

**DOI:** 10.3390/toxics14090833

**Published:** 2026-09-20

**Authors:** Zeynep Kilinc, Gamze Yesilay, Abdulkarim Rashed, Reem AlMealla, Batool Ahmed, Sakina Mustafa, Ahmed Khamis, Khalil Abdulla, Layla Hazeem

**Affiliations:** 1 Department of Molecular Biology and Genetics, University of Health Sciences-Turkey, Istanbul 34668, Türkiye; zeynepkilincsbu@gmail.com; 2 Experimental Medicine Application & Research Center, University of Health Sciences-Turkey, Validebag Research Park, Istanbul 34662, Türkiye; 3 Independent Researcher, Manama 18165, Bahrain; kme2001@hotmail.com; 4 Nuwat for Environmental Research & Education, Al Janabiyah 3050, Bahrain; reem@nuwat.org; 5 Department of Biology, College of Science, University of Bahrain, Sakhir P.O. Box 32038, Bahrain; bahmed@uob.edu.bh (B.A.); smustafa@uob.edu.bh (S.M.); 6 Supreme Council for the Environment (SCE), Manama P.O. Box 18233, Bahrain; askhamis@sce.gov.bh (A.K.); kali@sce.gov.bh (K.A.)

**Keywords:** microplastics, nylon-6, Raman spectroscopy, sub-10 µm particles, density separation, sediment pollution, Arabian Gulf, potential ecological risk index

## Abstract

Mangrove ecosystems are ecologically important coastal habitats, yet data on microplastic (MP) contamination along Bahrain’s coasts remain scarce. This study investigated the abundance, characteristics, and ecological risks of MPs in sediments from four mangrove sites (12 stations) collected in 2025. MPs were extracted via density separation and characterized by size, shape, color, and polymer composition using Raman spectroscopy; ecological risk was assessed using the Pollution Load Index, Polymer Hazard Index, and Potential Ecological Risk Index. MPs were detected at all stations, with abundances ranging from 533.33 ± 2.08 to 958.33 ± 6.5 particles/kg dry weight. Fragments were dominant in shape, and black was the dominant color. Particles < 10 μm and 10–30 μm were the most abundant size classes, with the sub-10 μm fraction, uncharacterized in Gulf mangrove ecosystems, suggesting that current methods underestimate MP abundance and associated ecological risk. Given their high surface reactivity, adsorption capacity, and potential root uptake, these ultrafine particles highlight the need for standardized, high resolution detection methods. Nylon-6 predominated all sampling sites, irrespective of their onshore or offshore setting, indicating common and persistent anthropogenic sources. Although ecological risk indices suggested an overall low-to-moderate environmental risk, the ubiquitous occurrence of MPs throughout Bahrain’s mangrove sediments raises concern regarding their long-term ecological implication. These findings provide a robust baseline for future spatiotemporal monitoring, refined ecological risk assessments, and the formation of targeted management strategies to safeguard coastal ecosystems.

## 1. Introduction

### 1.1. Microplastic Sources, Environmental Transport, and Ecological Threats

Microplastics (MPs) pollution has emerged as a global environmental challenge with far-reaching consequences for ecosystem integrity, food security, and human health. MPs, commonly defined as plastic particles less than 5 mm in diameter [1], originate from both primary and secondary sources. Primary MPs are intentionally manufactured at microscopic sizes for applications such as personal care products, industrial abrasives, and the manufacture of synthetic textiles, whereas secondary MPs are generated through the progressive fragmentation of larger plastic debris via ultraviolet (UV) radiation, mechanical abrasion, and physiochemical weathering processes [2,3,4]. Owing to their persistence and widespread dispersal, MPs have become ubiquitous across marine, freshwater, terrestrial, and atmospheric environments where they pose increasing risks to biodiversity, ecosystem functioning, and human health through multiple exposure pathways [3,5,6]. The environmental distribution of MPs is influenced by anthropogenic activities, hydrodynamic conditions, and geographical factors [6]. The ecotoxicological impacts of MPs include physical and chemical effects on aquatic organisms, bioaccumulation, and trophic transfer [2]. These particles can act as vectors for persistent organic pollutants and trace metals, causing physical harm and disrupting ecosystems, such as mangrove ecosystems [2,6,7]. Since MPs are readily transported through rivers, wastewater discharges, atmospheric deposition, and coastal currents, depositional environments such as salt marshes, estuaries, and mangrove forests often act as accumulation hotspots. Hence, these ecosystems are increasingly recognized as important sinks for MPs and valuable environments for investigating their distribution, retention, and potential ecological effects.

### 1.2. Mangroves as Multifunctional Coastal Ecosystems and Sinks for Microplastics

The structural and hydrodynamic characteristics that make mangroves highly effective at trapping sediments and organic matter also make them efficient sinks for MPs. Their complex aerial root systems reduce water flow velocities and enhance the deposition of fine sediments, facilitating the retention and accumulation of plastic particles transported from terrestrial and marine sources [8,9]. Consequently, mangrove sediments frequently contain higher concentrations of MPs than adjacent coastal habitats such as seagrass beds, saltmarshes, and unvegetated sediments [9,10]. Globally, MP concentrations reported in mangrove sediments vary substantially ranging from 1.22 to 6390 particles kg^−1^ dry weight [8,9], with the elevated levels commonly observed near urban centers, industrial zones, and fishing areas [10]. Most retained MPs are derived from land-based sources, including urban wastewater, stormwater runoff, and improperly managed plastic waste with fibers, fragments, polyethylene (PE), polypropylene (PP), and polystyrene (PS) frequently reported as dominant forms [8,9,10].

### 1.3. Regional Context: Mangroves in the Arabian Gulf and Bahrain

Mangrove stands in the Arabian Gulf exist in some of the most extreme environmental conditions experienced by mangrove ecosystems globally, including high salinity, elevated temperatures, and limited freshwater input [11,12]. These ecosystems are largely dominated by *Avicennia marina*, which is well adapted to the harsh conditions of the region. Despite their relatively limited geographic extent, Gulf mangroves provide important ecosystem services including shoreline stabilization, carbon sequestration, biodiversity support, and nursery habitats for commercially and ecologically important species.

Bahrain supports one of the smallest mangrove areas in the Gulf and has experienced substantial habitat loss over recent decades because of coastal reclamation, urban expansion, and associated pressures [13]. Aljenaid et al. (2022) reported that Bahrain has lost more than 95% of its natural mangrove cover between 1967 and 2020, declining to just 48 ha [14].

In response, Bahrain has prioritized mangrove conservation and restoration as part of its broader climate change mitigation and biodiversity strategies. National initiatives aim to quadruple the kingdom’s mangrove coverage by 2035 through large-scale restoration, afforestation, and habitat rehabilitation progress [13,15]. As a result of these initiatives, present-day mangrove stands in Bahrain comprise both naturally established stands, which developed through unassisted recruitment and growth, and afforested stands, established through active planting under restoration programs. This distinction is widely used in the mangrove restoration literature to differentiate the ecological status of restored versus undisturbed systems, since restored stands can initially differ from natural stands in structural, sedimentary, and biogeochemical attributes before converging toward comparable levels of maturity over time [16]. However, the timescale and extent of this convergence vary considerably across ecosystems, and whether a given classification of “natural” versus “afforested” continued to reflect a functionally distinct ecological state depends strongly on the stand age and site-specific conditions. In this study, we examine MP contamination across both natural and afforested mangrove stands in Bahrain to assess whether this classification is reflected in observed pollution patterns.

### 1.4. Aims of the Study

This study aims to (1) quantify the abundance of MPs in sediments collected from natural and afforested mangrove stands in selected sites in Bahrain. (2) Characterize MPs according to their size, shape, color, and polymer composition in the sediments. (3) Compare MP abundance, size fractions, and polymer compositions between onshore and offshore mangrove-associated sites to evaluate spatial patterns of contamination. (4) Assess the potential ecological risk associated with MP contamination using standardized indices: Pollution Load Index (PLI), Polymer Hazard Index (PHI), and Potential Ecological Risk Index (RI).

## 2. Materials and Methods

### 2.1. Study Area

**Figure 1:**
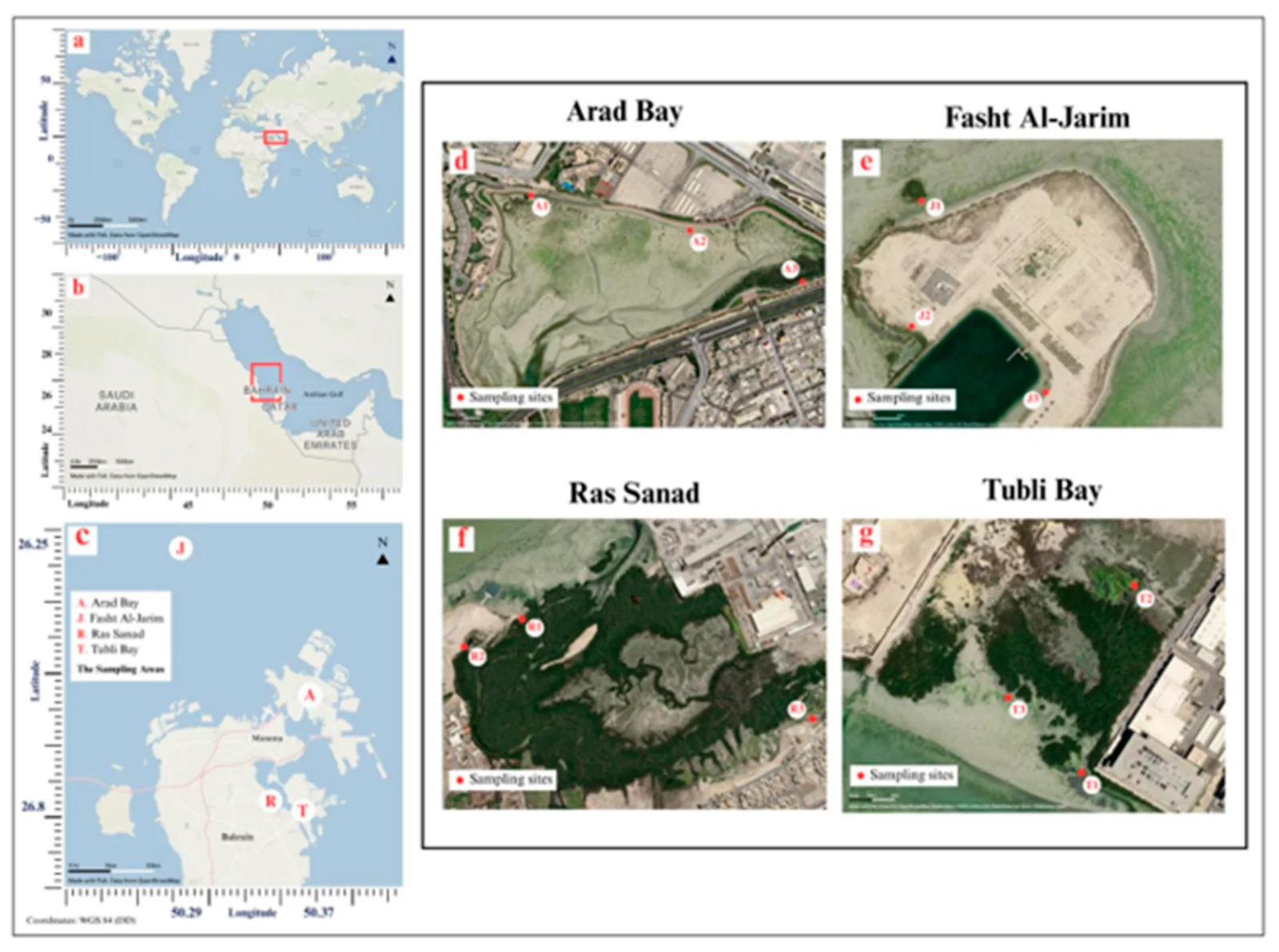
Locations of study area: (**a**) location of Arabian Gulf (AG); (**b**) location of Bahrain within the AG; (**c**) location of selected study sites within Bahrain; (**d**) Arad Bay; (**e**) Fasht Al-Jarim; (**f**) Ras Sanad; and (**g**) Tubli Bay.

The study was carried out in four coastal areas: two inshore areas (Tubli Bay, which includes Ras Sanad and Arad Bay) and one offshore (Fasht Al-Jarim) (Figure 1). Tubli Bay is located in the northeast of Bahrain and is characterized by a sheltered environment that supports diverse habitats such as mudflats, algal mats, and mangrove swamps [13]. Given its significance as a major feeding site for large populations of wading birds, the area was designated a Ramsar site in 1997 [13]. Tubli Bay contains significant natural mangrove stands in Bahrain, primarily concentrated in the Ras Sanad region [13]. The muddy sediment characteristics along with the historically abundant inflow of low-salinity water from nearby farms created favorable conditions for mangrove growth and development [17]. Historically, mangroves were widespread along the coastline of Tubli Bay [18].

Ras Sanad, located within Tubli Bay, contains Bahrain’s largest remaining natural mangrove stand. Although the site has been managed for conservation purposes since 1988, it was formally designated as a Category A protected area under Ministerial Order No. 1 of 1995, where reclamation and construction are prohibited [19]. Protection was subsequently reinforced by Law No. (53) of 2006, designating Tubli Bay as a Natural Protected Area [17,20].

Arad Bay, situated in the northeast of Bahrain, is a sheltered mudflat covering approximately 0.5 Km^2^ and supports an extensive intertidal zone rich in benthic fauna [21]. The Bay was declared a natural marine protected area in 2003 [22] due to its value as a foraging and roosting site for key shorebird communities [23]. Besides its ecological importance, Arad Bay also serves as a nature reserve offering recreational and public services. Mangrove transplantation efforts have been ongoing there since 1998, contributing to increased coastal productivity through enhancing macrobenthos and bird diversity [18,23].

Fasht Al-Jarim is a subtidal area, located 20 Km off Bahrain’s north coast, has historically supported diverse marine ecosystems, including coral reefs and seagrass beds providing nursery habitats for marine organisms [21,24,25], though its coral reef ecosystem has since degraded alongside a shift toward algal dominance [26,27]. Mangroves here can support coral reef resilience by providing nursery habitats for reef-associated fish and invertebrates [28]; both ecosystems remain exposed to threats including coastal reclamation, elevated sea temperatures, sedimentation, and pollution, constraining long-term ecosystem recovery [21,25,27,29].

### 2.2. Sediment Sample Collection

For this study, sediment samples were collected during low tide from four mangrove sites dominated by *Avicennia marina*, with three different stations at each sampling site (Table 1). As naturally recruited stands, Ras Sanad and Tubli Bay have no documented establishment or planting date; however, given their historical presence along the Tubli Bay coastline and their protected status since the 1980s–1990s [17,19], both are considered long-established, mature natural stands, in contrast to the more recently established afforested sites (Arad Bay, ~27 years; Fasht Al-Jarim, ~12 years). Each station’s proximity to the nearest road was qualitatively assessed from station coordinates (Table 1). Most onshore stations (Arad Bay A1–A3, Tubli Bay T1, Ras Sanad R2–R3) were situated in close proximity to a road (<50 m), while Tubli Bay T2 and T3 and Ras Sanad R1 were located at a moderate distance from the nearest roadway (~100–150 m). The offshore Fasht Al-Jarim site, located approximately 20 km from the mainland, has no adjacent road infrastructure, and this criterion was not applicable. Sampling was conducted in the period January–June 2025. The sites were selected to represent a range of environmental settings and anthropogenic pressures such as proximity to the mainland, fishing activities, and ecotourism areas as demonstrated in Table 1. At each mangrove site, three stations were selected, which were randomly spaced, to account for spatial variability (i.e., haphazard/opportunistic placement based on accessibility), and within 0.5 m of the nearest trunk base, holes were dug in bare sediment patches between pneumatophores to avoid root damage, with three replicate samples at each station from the upper 2–3 cm using 20 × 20 cm quadrats and stainless-steel shovel. The samples were collected in glass containers previously washed with deionized water and covered with aluminum foil. The samples were transferred to the laboratory and stored at −20 °C until the day of processing. The geographical location and characteristics of each mangrove site are presented in Figure 1 and Table 1. These sediment samples were used exclusively for MP extraction and were independent of the sediment samples collected for grain size analysis and the water samples collected for the physicochemical and nutrient analysis (Section 2.5).

### 2.3. Laboratory Processing and Microplastic Extraction

The sediment samples were first left to thaw at room temperature for at least 24 h before processing. After thawing, the samples were dried in an oven (LTE Scientific, Oldham, UK) at 60 °C for a minimum 24 h. To avoid contamination, this oven was dedicated solely to drying materials used in MP analysis, with airflow kept to a minimum throughout the procedure.

The extraction procedure for MPs was modified from the methods outlined by Jahan et al. (2019), Zhange et al. (2021), and Hammadi et al. (2022) [30,31,32]. A density separation approach was used to separate low-density MP particles from the denser mineral and organic components. For this process, 40 g of dried sediment was combined with 75 mL of filtered saturated NaI solution with a density of approximately 1.80 g cm^−3^ enabling the recovery of both low- and high-density MPs. The mixture was then stirred continuously for at least five minutes using a metal spoon, following the method outlined in Zhang et al. (2021) [31]. Each sample was covered with aluminum foil and left to settle for a minimum of 24 h or until the supernatant became clear. This settling period allowed the complete separation of the solid and liquid phases, ensuring that the solution stabilized and that the MPs floated to the surface of the supernatant [33]. Following the guidelines of Han et al. (2019), Hosseini et al. (2020), and Nyguen et al. (2020), the overflow procedure was then performed once the samples had fully settled [34,35,36]. The filtered saturated NaI solution was then utilized to perform the overflow procedure. Following this step, a 10% KOH solution was added to the supernatant in a 3:1 ratio (KOH: supernatant) of a 10% KOH solution, which was added to effectively break down and remove any remaining organic matter. The mixture was allowed to digest for a minimum of 24 h, after which it was subjected to vacuum filtrations using fiberglass filters (Whatman GF/F filter discs with a 0.7 µm pore size (WHA1825047), Maidstone, UK). The filters were subsequently stored in pre-washed glass containers and sealed with aluminum foil until further analysis.

### 2.4. Identification of Microplastics with Raman Spectroscopy

Raman spectra of MPs were obtained using a confocal Raman Microscope (Renishaw, inVia Qontor, Wotton-under-Edge, Gloucestershire, UK). A spectral range of 605–1715 cm^−1^ was scanned with a 785 nm wavelength laser at 100 mW laser power, with 1200 lines/mm grating and a 5 s laser exposure time in StreamHR scanning mode with a 1 cm^−1^ spectral resolution.

The spectral data were processed using WiRE 5.7 software, including cosmic ray removal and baseline subtraction. Polymer types of MPs were assigned by comparing the obtained Raman fingerprint spectra with reference spectra reported in the literature [37,38,39,40,41,42,43,44,45,46,47,48,49,50,51].

### 2.5. Physicochemical Water Quality and Sediment Size Analysis

Physicochemical water quality parameters were measured in situ at each sampling site, within the same sampling area, but independently of the sediment sampling conducted for MP analysis (Section 2.2), using a multiparameter water quality meter (ProDSS, YSI Inc., Yellow springs, OH, USA).

Water samples for nutrient analysis were collected in pre-cleaned PE bottles at each sampling site and transported to the laboratory for analysis. Ammonia, phosphate, and nitrate concentrations were determined in triplicate using a photometer (Palintest 7100, Palintest Ltd., Gateshead, UK), following the manufacturer’s standard procedures, in which ammonia was detected using the indophenol blue method (0–1 mg/L NH_3_-N), phosphate using phosphomolybdenum blue (0–4 mg/L PO_4_), and nitrate using the diazonium reaction (0–20 mg/L NO_3_-N). These water samples were collected within the same sampling area, but independently of the sediment samples used for MP extraction (Section 2.2).

Sediment grain size analysis was conducted to characterize the sediment texture at each sampling site. Three sediment subsamples were collected from each sampling site. Approximately 40 g of each subsample was oven-dried at 105 °C for 24 h to obtain a constant dry weight. The dried sediments were then subjected to dry sieve analysis using a stack of standard sieved with mesh sizes ranging from 4 mm to 0.063 mm. The sediment retained on each sieve was weighed, and the proportion of each grain-size fraction was expressed as a percentage of the total dry weight. Grain-size classes were classified according to the Wentworth scale [52]. The fraction passing through the 0.063 mm sieve was reported as the fine fraction (silt and clay).

### 2.6. Ecological Risk Assessment

Ecological risk assessment of MPs was conducted based on their abundance and polymer composition using the Pollution Load Index (PLI), Polymer Hazard Index (PHI), and Potential Ecological Risk Index (RI), following the methodology described by Kor et al. (2026) [53]. Due to the unavailability of hazard scores for all detected polymer types, PHI and RI calculations were limited to polymers with defined hazard values, including poly (ethylene terephthalate) (PET), PP, PS, polyurethane (PU), and Nylon-6 [54].

### 2.7. Quality Assurance and Quality Control

To minimize the risk of sample contamination, strict quality assurance and quality control (QA/QC) measures were implemented throughout sample collection and laboratory analysis. All procedures were carried out under a fume hood, and work surfaces were routinely cleaned with ethanol and deionized water before use. We acknowledge that the deionized water used for equipment and container cleaning was not passed through an additional particle filter (e.g., 0.2–0.45 µm) prior to use and therefore cannot be assumed free of trace MP particles, particularly the sub-10 µm size range emphasized in this study’s findings. To account for this potential contamination pathway, field and laboratory blanks were processed alongside the environmental samples (see below), providing an empirical, rather than assumed, estimate of procedural background. The use of plastic materials was avoided during sampling, and laboratory analyses were performed under a cotton cover to reduce the risk of airborne fiber contamination [55]. All analytical and sampling equipment was thoroughly cleaned with filtered deionized water and rinsed with 70% ethanol prior to use, and glass containers were sealed with aluminum foil to prevent contamination during storage.

To evaluate the extraction efficiency, PS was used as a positive control. To assess potential background contamination, two field blanks and two laboratory blanks were incorporated into the analytical workflow as negative controls: field blanks were opened and exposed at the sampling site during sample collection to capture contamination introduced in the field, while laboratory blanks were processed alongside the environmental samples during extraction and analysis to evaluate contamination arising during handling and preparation in the laboratory. As these blanks were not collected at every sampling station or run with every laboratory processing batch, they provide a general estimate of procedural background contamination rather than a station- or batch-specific correction.

### 2.8. Data Analysis

All data processing, statistical analyses, and visualizations were conducted in RStudio (version 4.5.1) [56]. Chord diagrams were generated using R package [57] following the standard circlize convention in which arc length reflects category totals and ribbon width reflects pairwise co-occurrence frequency. The sizes of MPs were measured using ImageJ Software (version 1.54g).

One-way analysis of variance (ANOVA) was employed to evaluate differences in MP abundance among sampling sites. Prior to analysis, the assumptions of normality and homogeneity of variances were assessed using the Shapiro–Wilk and Levene’s tests, respectively. When these assumptions were not met, data were subjected to square-root transformation. Spearman correlation analysis was employed to evaluate the relationship between ecological risk matrices.

Permutational multivariate analysis of variance (PERMANOVA; Adonis function in R [58]) based on Bray–Curtis distance matrices was used to test for differences in polymer compositions between sampling groups (onshore vs. offshore and natural vs. transplanted mangrove sites). Similarity percentage (SIMPER) analysis was performed to identify the polymers contributing most across sampling groups [59]. A significance level of *p* < 0.05 was used for all statistical tests.

Principle Component Analysis (PCA) was applied to examine the association between MP abundance and physicochemical parameters (ammonia, phosphate, nitrate, temperature, dissolved oxygen (DO), salinity, and pH) [60]. Before analysis, the dataset was log-transformed and standardized to minimize the influence of scale differences among variables.

## 3. Results

### 3.1. Sampling Overview and Sediment Characteristics

MPs collected from all mangrove sampling sites were identified using Raman spectroscopy, and their polymer types were assigned based on comparisons with reference spectra reported in the literature [37,38,39,40,41,42,43,44,45,46,47,48,49,50,51] (Figure S1, Table S1). In total, ten types of plastics were detected across all mangrove sampling sites (Figure S1), namely copper phthalocyanine-PP (Copper phthalocyanine-PP), fluorinated ethylene propylene (FEP), PET/diamine/multi-walled carbon nanotube (MWCNT) composites (PET/diamine/MWCNT), PS, polytetrafluoroethylene (PTFE), and PU. Based on our findings, the most frequently observed MP shape and color were black fragments.

Raman analysis of the PS positive control produced characteristic spectra consistent with the reference PS fingerprint, confirming the reliability of spectral acquisition and polymer identification (Figure S2).

To assess potential procedural contamination, two laboratory blank filters and two field blank filters were processed and analyzed alongside the environmental samples. A total of 49 Raman measurements were collected from the laboratory blanks, of which 14 (28.6%) corresponded to synthetic particles, 7 particles were identified as PET/diamine/MWCNT composite, 4 as copper phthalocyanine-PP, 1 as PS, and 2 as unidentified spectra, while the remaining 35 spectra (71.4%) were classified as spectral noise. For the field blank filters, 132 Raman spectra were acquired, of which 24 (18.2%) corresponded to synthetic particles, 10 particles were identified as PET/diamine/MWCNT composite, 1 as PS, 1 as disordered graphene, and 12 as unidentified spectra, whereas the remaining 108 spectra (81.8%) were classified as spectral noise. Notably, none of the polymer types detected in either blank type matched Nylon-6, the dominant polymer identified in the environmental samples, indicating that laboratory or field contamination is unlikely to account for the main polymer composition findings. Because blank filters were processed per sampling batch rather than paired individually with each sample or station, a particle-by-particle blank correction could not be reliably applied to the environmental counts. Blank contamination levels are therefore reported here as a transparency measure and as an upper-bound estimate of procedural background, and the reported MP abundance should be interpreted with this background in mind. We recommend that future surveys incorporate station-paired blanks to enable direct quantitative correction.

### 3.2. Microplastic Abundance and Size Distribution

**Figure 2:**
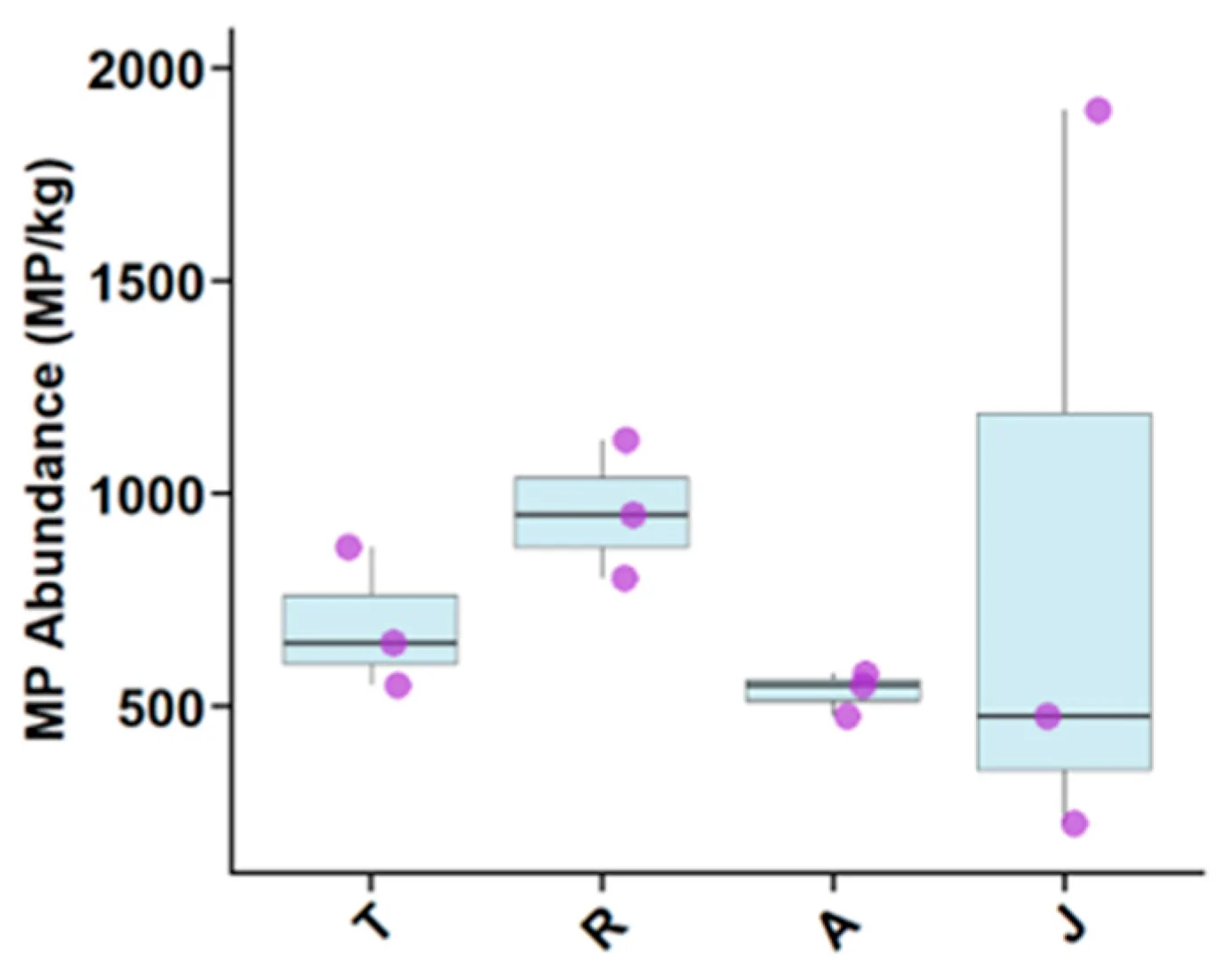
Abundances of MPs expressed as MP particles per kilogram in mangrove samples collected from different study sites. Each dot represents an abundance of MPs across sampling sites. Boxplots represent the first and third quartiles, with the central line indicating the median. Whiskers indicate the most extreme data point that is lower than 1.5 times the interquartile range from the box [T: Tubli Bay; R: Ras Sanad; A: Arad Bay; J: Fasht Al-Jarim].

MP abundance varied markedly among mangrove sites, spanning from 533.33 ± 2.08 to 958.33 ± 6.5 MP/kg d.w. (Figure 2). Across natural mangrove sites (T1–T3 and R1–R3), abundances ranged between 550 MP/kg d.w. (T2) and 1125 MP/kg d.w. (R2), with intermediate values at T1 (650 MP/kg d.w.), T3 (875 MP/kg d.w.), R1 (800 MP/kg d.w.), and R3 (950 MP/kg d.w.). In afforested mangrove sites (A1–A3 and J1–J3), MP abundance exhibited a wider spread, from 225 MP/kg d.w. (J1) to 1900 MP/kg d.w. (J2), while the remaining sites showed moderate and relatively comparable levels (A1: 575; A2: 475; A3: 550; J3: 475 MP/kg d.w.). MP abundance did not differ significantly between natural and afforested mangrove sites (F = 0.723, *p* = 0.415).

MP abundances at onshore sites (T1–T3, R1–R3, and A1–A3) ranged from 475 MP/kg d.w. (A2) to 1125 MP/kg d.w. (R2), whereas MP abundances at offshore sites (J1–J3) were observed to range between 225 MP/kg d.w. (J1) and 1900 MP/kg d.w. (J2). No significant difference was found in MP abundance among shoreline mangrove sites (F ≈ 0, *p* = 0.987).

**Figure 3:**
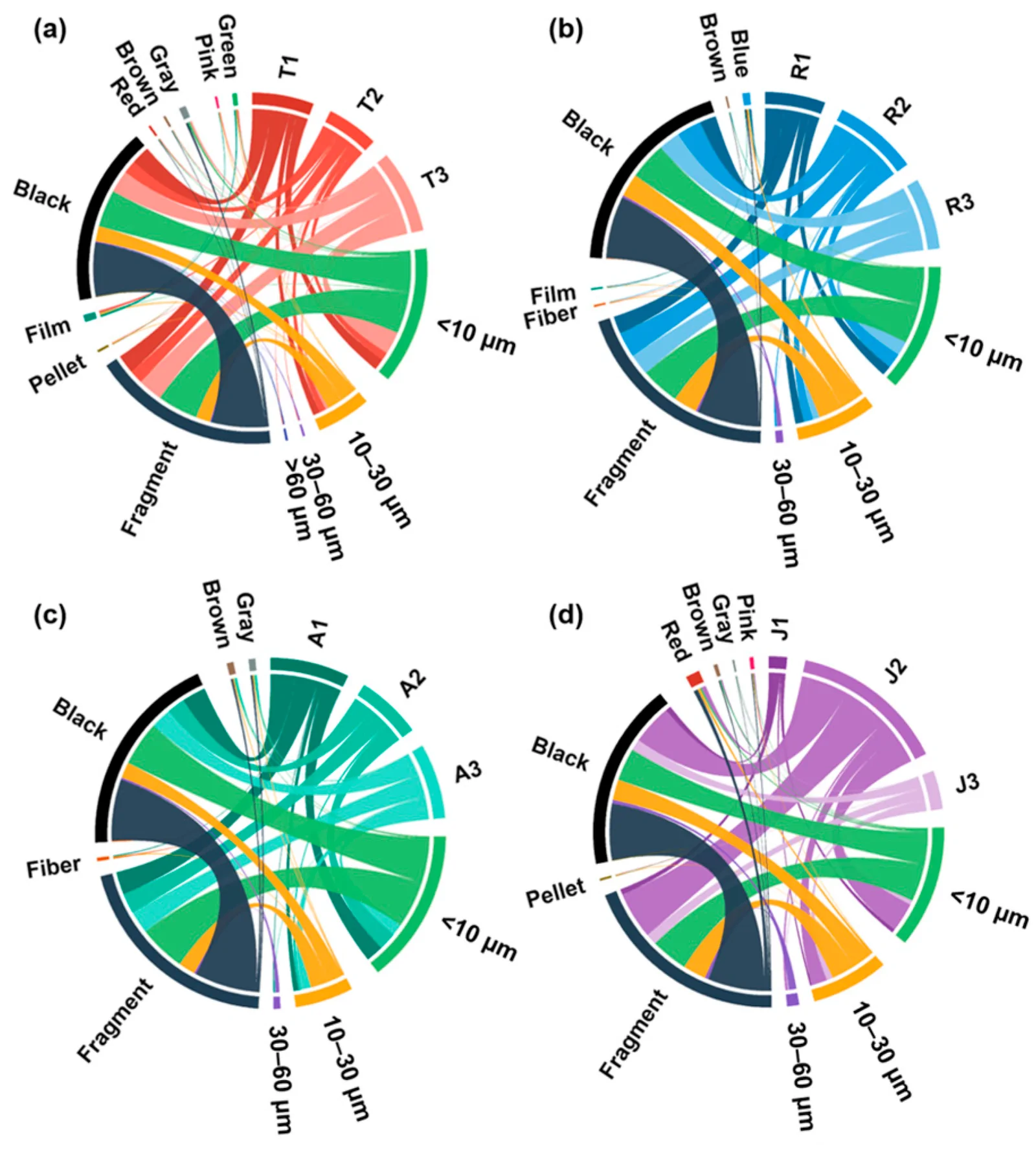
Chord diagrams of MP sizes, shapes, and colors across mangrove sampling sites: (**a**) Tubli Bay (T1–T3) (*n* = 83), (**b**) Ras Sanad (R1–R3) (*n* = 115), (**c**) Arad Bay (A1–A3) (*n* = 64), and (**d**) Fasht Al-Jarim (J1–J3) (*n* = 104). Each diagram is organized into four arc groups arranged around the circle—sampling station, MP color, MP shape, and MP size class—with the length of each arc proportional to the total number of particles in that category. Ribbons connecting two arcs indicate the co-occurrence of the corresponding categories within the same sample (for example, a ribbon linking “T2” to “Black” indicated black-colored particles recovered at station T2); the ribbon width is proportional to the frequency (count, n) of that co-occurrence, and ribbon color follows the linked source category to aid visual tracing. Thicker ribbons therefore denote more frequent associations between categories, allowing multiple categorial relationships to be compared within a single panel.

The particle size distribution of MPs across all regions and samples ranged from 1.1 to 66.1 µm, with most particles within the <10 µm and 10–30 µm size classes. Therefore, MPs were grouped into four size classes: <10 µm, 10–30 µm, 30–60 µm, and >60 µm (Figure 3). At natural mangrove sites (T1–T3 and R1–R3), particles spanned the full-size spectrum (~1.1–66.1 µm), whereas at afforested mangrove sites (A1–A3 and J1–J3), they were confined to all size classes except >60 µm (~2.0–57.4 µm). A similar pattern was observed along the onshore—offshore gradient: MPs at onshore sites (T1–T3, R1–R3, and A1–A3) covered the entire size range (~1.1–66.1 µm), while those at offshore sites (J1–J3) were restricted to all classes below 60 µm (~2.0–57.4 µm).

MPs were classified into four shapes: fibers, fragments, films, and pellets. Fragments (97.8%) were the most common MP shape, followed by films (1.09%), fibers (0.55%), and pellets (0.55%), across all mangrove sites (Figure 3). In terms of color, black was the most dominant MP color (92.1%), followed by red (2.19%), brown (1.64%), gray (1.64%), blue (1.09%), pink (0.82%), and green (0.55%) (Figure 3).

### 3.3. Polymer Composition and MP Types

**Figure 4:**
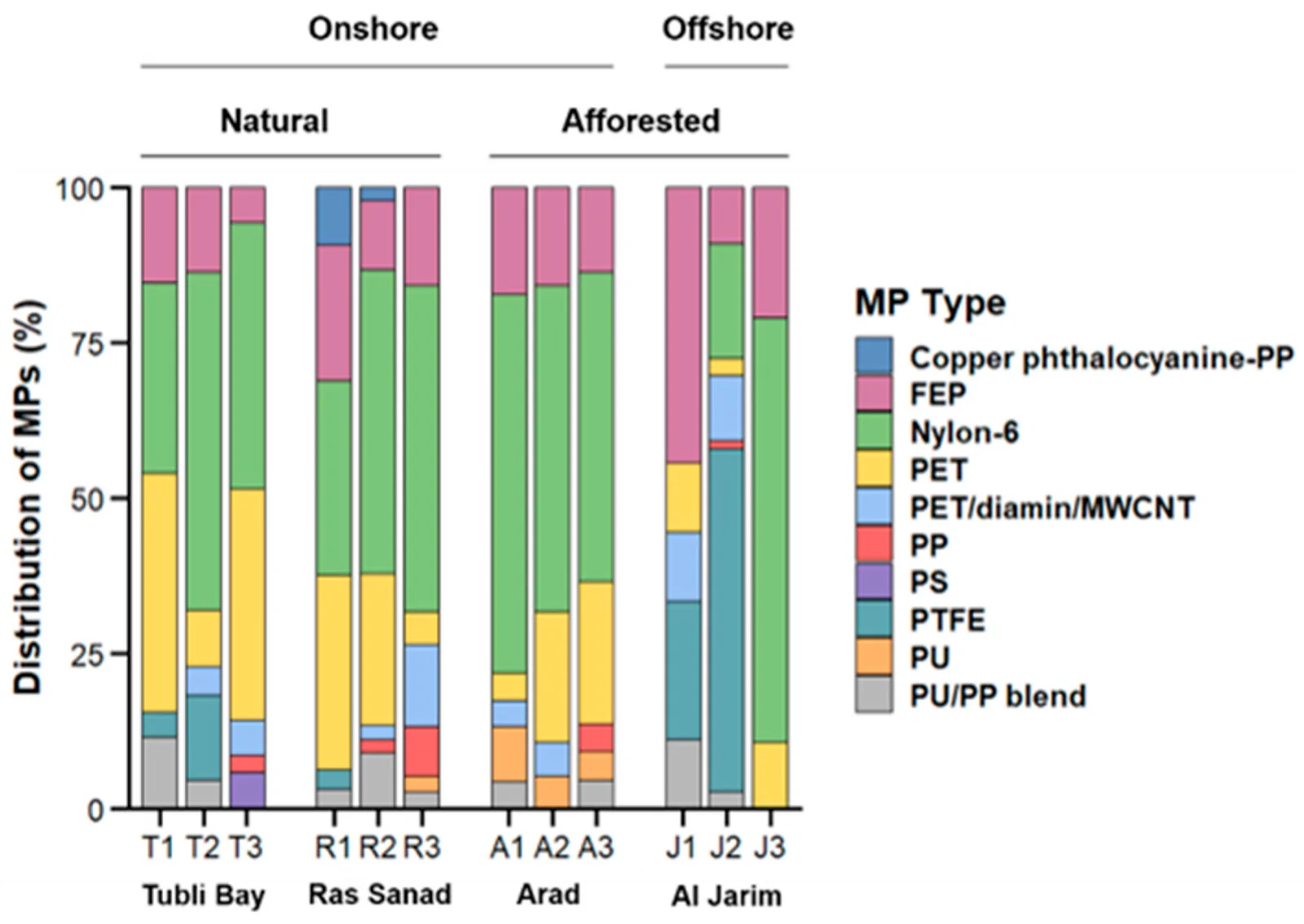
Distribution of MP types in mangrove samples across the study sites (natural/afforested and onshore/offshore). The MPs include copper phtalocyanine-PP, FEP, Nylon-6, PET, PET/diamine/MWCNT composite, PP, PS, PTFE, PU, and PU/PP blend.

Nylon-6, PET, and FEP were the most frequently observed MP types across all sites. Onshore and natural mangrove sites were dominated by Nylon-6 and PET, whereas afforested and offshore mangrove sites mainly composed of Nylon-6, PET, FEP, and PTFE, as illustrated in Figure 4.

Nylon-6 dominated the polymer composition at both onshore and offshore locations. At onshore sites (T1–T3, R1–R3, and A1–A3), it accounted for 30–61% of the detected MPs. At offshore sites (J1–J3), their contribution was even higher, reaching 68.5%. When sites were further grouped by mangrove type, Nylon-6 remained a major component in both categories: at natural mangrove sites (T1–T3 and R1–R3), its relative abundance ranged from 31% to 54.5%, whereas at afforested sites (A1–A3 and J1–J3), it showed consistently elevated proportions, reaching a maximum of 68.5%.

FEP was detected in all sites and was particularly prominent at offshore sites; 44.4% of FEP was found at J1, whereas 22% of FEP was detected at the onshore sites (R1). FEP distribution ranged from 6% to 16% and from 9% to 44.4% at natural and afforested mangrove sites, respectively.

PTFE was observed at only offshore sites, with a distribution level of 22.2% in J1 and 55.3% in J2, whereas it was either absent or present at very low levels at other shoreline mangrove sites.

Additional MP types, including PET/diamin/MWCNT composites, PP, PU, and PU/PP blends, were observed across several sites, however, generally at lower distribution levels. Copper phthalocyanine-PP was detected only at R1 and R2, whereas PS was observed at only T3.

PET ranged from 4% to 38.5% and from 3% to 11.1% at onshore and offshore sites, respectively. The highest PET distribution was observed in natural site T1 (38,5%), whereas the lowest value (3%) was recorded at the afforested site J2. The near-significant PERMANOVA result for shoreline sites (*p* = 0.069) indicated variation in the MP composition; however, SIMPER analysis did not identify any significant polymer contributing to this dissimilarity. In contrast, no significant differences were found between natural and afforested mangroves (PERMANOVA, *p* = 0.201), whereas SIMPER identified PET as a significant contributor (*p* = 0.036).

### 3.4. Comparative Analyses

**Figure 5:**
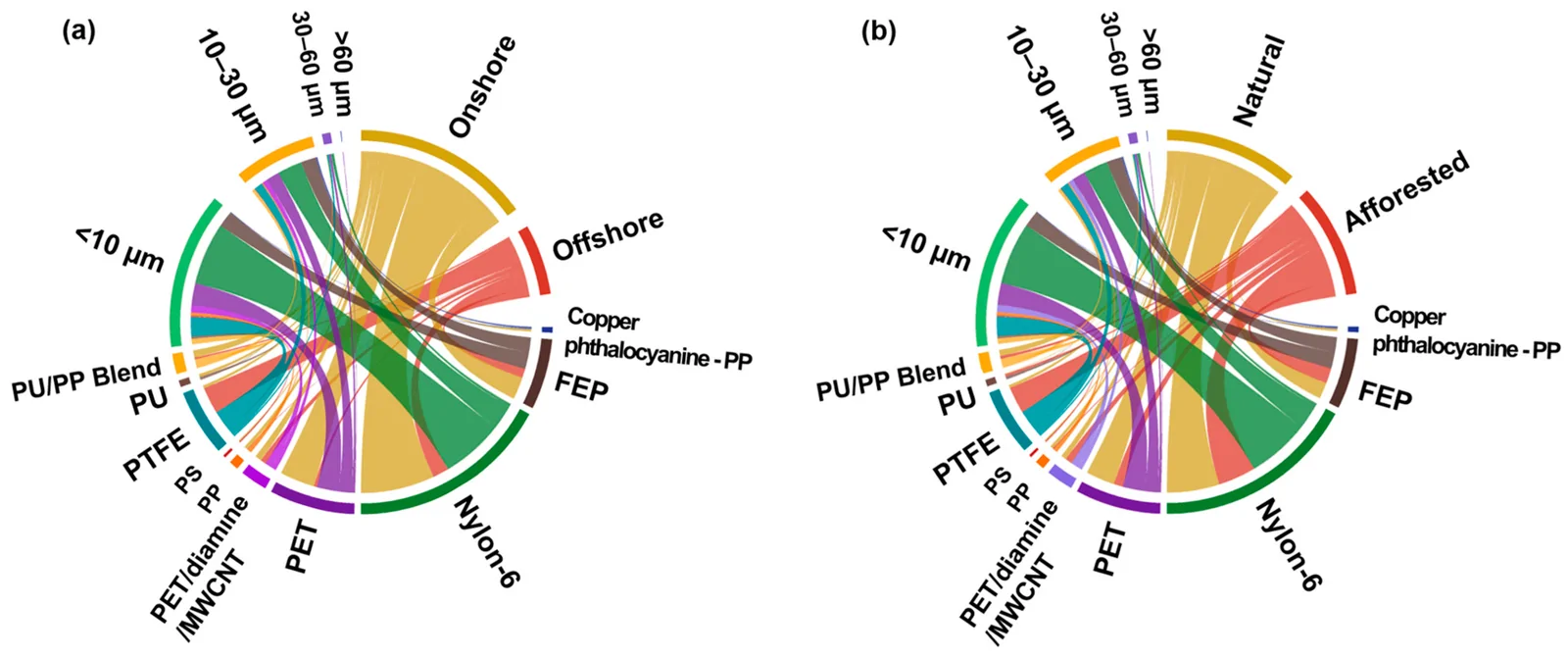
Chord diagrams of MP sizes and types across mangrove sampling sites. (**a**) Onshore/offshore and (**b**) natural/afforested. The MPs include copper phthalocyanine-PP, FEP, Nylon-6, PET, PET/diamine/MWCNT composite, PP, PS, PTFE, PU, and PU/PP blend. Each diagram is divided into arc groups (MP types, MP size class, and mangrove sampling sites (onshore/offshore or natural/afforested)) positioned around the circle, with the arc length proportional to the total count of particles in each category. Ribbons connecting two arcs represent the co-occurrence of the linked categories (for example, a ribbon between “Nylon-6” and “offshore” indicated Nylon-6 particles detected at offshore sites), with the ribbon width scaled to the frequency (count, n) of that co-occurrence and ribbon color following the source category.

Figure 5 illustrates the relationship between MP size classes and the distribution of suggested MP types across mangrove sampling sites (onshore/offshore and natural/afforested). MPs were predominantly observed within the <10 µm and 10–30 µm size fractions, which were associated with polymer types, including Nylon-6, PET, FEP, and PTFE across mangrove sampling sites.

Onshore and natural sites exhibited a broader and more diverse distribution of MP types across size classes, with Nylon-6 primarily concentrated in particles <30 µm and PET occurring across the full-size spectrum (Figure 5a,b). In contrast, offshore and afforested sites were characterized by a more restricted polymer assemblage, dominated by FEP, PTFE, and Nylon-6, which were mainly associated with particles below 60 µm (Figure 5a,b).

PCA was performed to evaluate the relationship between MP abundance and the physicochemical parameters across the sampling locations. PC1 explained 34.75% of the total variance, while PC2 and PC3 explained 20.49% and 17.34%, respectively, resulting in a cumulative variance of 72.58% for the first three components. PC1 was characterized by DO (−0.546), pH (−0.491), and temperature (−0.421), which were inversely correlated with salinity (0.334). In PC2, nitrate (0.557), MP abundance (0.449), and phosphate (0.314) were directly correlated, whereas ammonia (−0.476) was inversely related to these variables. In PC3, phosphate (−0.670), ammonia (−0.534), and salinity (−0.403) were directly correlated. The PCA plot is provided in the Supplementary Material (Figure S3) and illustrates the distribution of sampling sites in relation to physicochemical parameters and MP abundance. Detailed physicochemical and nutrient concentration data for each sampling site are presented in the Supplementary Material (Table S2).

Grain size analysis (Table 2) indicated that sediment at all sampling sites was predominately sandy (63.2–89.5%), with the finer silt + clay fraction ranging from 2.8% (J3) to 35.4% (J2). Arad Bay and Fasht Al-Jarim stations generally showed higher proportion of fine sediment than Tubli Bay and Ras Sanad, although sand remained the dominant fraction throughout.

### 3.5. Ecological Risk

**Figure 6:**
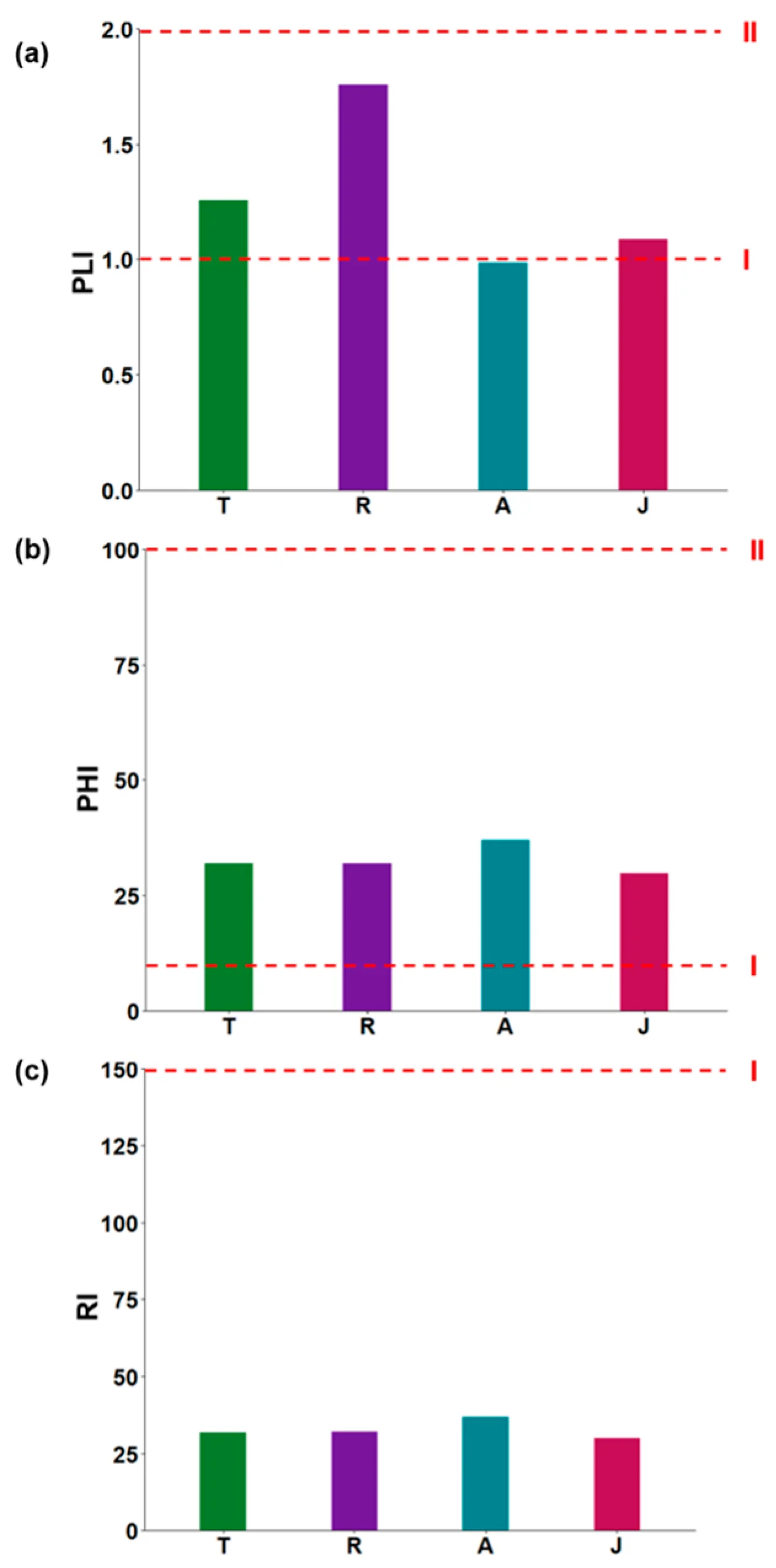
Ecological risk ((**a**) PLI, (**b**) PHI, (**c**) RI) of MPs among different mangrove sites [T: Tubli Bay; R: Ras Sanad; A: Arad Bay; J: Fasht Al-Jarim].

The ecological risks caused by MPs in the collected mangrove sediments were evaluated using three different indices: PLI, PHI, and RI (Figure 6 Tables S3 and S4). PLI values for Tubli Bay (T), Ras Sanad (R), Arad Bay (A), and Fasht Al-Jarim (J) regions were 1.26 ± 0.31, 1.76 ± 0.30, 0.98 ± 0.10, and 1.09 ± 1.67, respectively; 25% of the sampling sites were classified as risk level I, while 75% were risk level II. PHI values for the T, R, A, and J regions were 31.90 ± 10.12, 31.92 ± 7.94, 36.95 ± 5.46, and 29.86 ± 22.42, respectively. According to the risk classification, all sampling sites were categorized under risk level II. RI values for the T, R, A, and J regions were 38.87 ± 8.81, 54.90 ± 19.52, 36.12 ± 8.00, and 21.13 ± 75.40, respectively. Based on this index, all sampling sites were classified as risk level I. In this study, the correlation between RI and PLI (R^2^ = 0.60) was stronger than the correlation between RI and PHI (R^2^ = 0.30) (Figure S4).

## 4. Discussion

### 4.1. Key Findings

This study confirms the pervasive occurrence of MPs within mangrove ecosystems of the Kingdom of Bahrain, with MP contamination detected at all sampled sites, consistent with the global ubiquity of MP pollution in mangrove environments [8].

The offshore afforested site, Fasht Al-Jarim, exhibited a relatively high abundance of MPs (866.67 ± 36.14 MP/kg d.w.), with the greatest concentration recorded at station J2 (1900 MP/kg d.w), whereas substantially lower abundances were observed at station J1 (225 MP/kg d.w) and J3 (475 MP/kg d.w), respectively. The pronounced enrichment of MPs at station J2 warrants further investigation into the sources and transport pathways responsible for plastic accumulation in this offshore habitat. In the absence of significant anthropogenic activities nearby, the elevated MP concentrations are more likely attributable to regional transport processes, including tidal currents, hydrodynamic circulation, fishing-related activities, and other diffuse marine based inputs rather than direct local sources. These findings highlight the need for comprehensive studies integrating hydrodynamic modeling, source apportionment, and spatial monitoring to clarify the mechanisms governing MP transport and accumulation at Fasht Al-Jarim and to support effective management of offshore mangrove ecosystem.

Ras Sanad was the second mangrove site with a high level of MPs (958.33 ± 6.5 MP/kg d.w.), ranging from 1125 MP/kg d.w. at R2 to 800 MP/kg d.w. at R1. It is protected and a natural mangrove site with high density of mangrove trees. It is located within Tubli Bay, and it has a nursery ground of mangroves. The high concentration of MP pollution observed can be explained by the high land-based pollution inputs from nearby anthropogenic activities and high concentration of plastic waste along the sampling stations.

The third highest concentration of MPs was recorded in Tubli Bay (691.67 ± 6.6 MP/kg d.w.), which is also a protected area and natural mangrove site. The highest concentration was found at station T3 (875 ± 6.37 MP/kg d.w.), followed by 650 ± 3.7 MP/kg d.w. and 550 ± 4.18 MP/kg d.w. at stations T1 and T2, respectively. The site-specific characteristics observed during field surveys may further contribute to the variability in MP concentrations across sampling stations. At T3, the area supports thousands of migratory and resident wading birds, which utilize the site as a resting ground during high tide and as both a feeding and resting area during low tide. Avifauna represents a potentially overlooked biological vector for MP introduction, as particles may adhere to plumage or be deposited through fecal matter, contributing to local MP loads. At T1, the site similarly functions as a feeding and resting area for birds during low tide, suggesting a comparable, albeit potentially lesser, biological input. Conversely, station T2, located approximately 150 m from a major roadway, experiences restricted hydrodynamic exchange and low water renewal rates. These conditions likely favor the retention and accumulation of MPs by limiting particle export while enhancing the deposition of contaminants derived from road runoff, atmospheric fallout, and other localized anthropogenic sources. Collectively, these observations demonstrate that MP distribution is controlled by interplay between regional inputs and site-specific hydrodynamic and ecological processes, which ultimately govern particle transport, deposition, and accumulation.

Sediment texture is widely recognized as a control for MP retention, as finer sediments offer greater surface area and lower permeability, favoring particle entrapment relative to coarser, more permeable sands [61]. Across the sampled stations, the silt + clay fraction showed a weak positive association with MP abundance (e.g., station J2, which recorded both the highest MP abundance and the highest fine-sediment content, 34.5%), while station J1, with the lowest fine-sediment content among offshore stations, also recorded the lowest MP abundance. However, given that sand remained the dominant fraction at every station and the relationship was not consistent across all sites (e.g., T3 combined high sand content with elevated MP abundance), grain size is best interpreted as one of the several contributing factors, alongside hydrodynamic and anthropogenic drivers, rather than the primary determinant of the spatial pattern observed in this study.

Land-based anthropogenic pressures contribute substantially to increase MP inputs through pathways such as wastewater discharge, urban surface run-off, and coastal land reclamation, particularly within semi-enclosed environments characterized by restricted water circulation. Furthermore, the degradation and fragmentation of the mangrove stand in Tubli Bay and Ras Sanad have likely diminished the ecosystem capacity to intercept and retain suspended particulate matter, thereby promoting the accumulation and long-term persistence of MPs in both sediments and adjacent surface waters. Accordingly, the elevated MP concentrations observed in Ras Sanad and Tubli Bay agree with the combined effects of anthropogenic activities and ecosystem degradation reported by Mohan et al. (2024), further supporting the conclusion that intensive coastal development together with mangrove habitat loss amplifies MP contamination in highly urbanized coastal embayment [62].

Although Ras Sanad and Tubli Bay are situated at similar distances from the Tubli Bay Sewage Treatment Plant and are exposed to comparable anthropogenic sources of MPs, they exhibited markedly different concentrations. This discrepancy indicates that spatial proximity to potential pollution sources alone cannot adequately account for the observed distribution of MPs in coastal ecosystems. Instead, local environmental factors, including hydrodynamic conditions, water circulation, sediment characteristics, and bathymetric features, are likely to exert a stronger influence on MP transport, retention, and redistribution. Therefore, attributing the MP concentration solely to geographic proximity should be approached with caution, as the interaction of multiple physical and environmental processes can substantially modify MP accumulation patterns irrespective of the source location. These observations highlight the importance of comprehensive, long-term monitoring and site-specific investigations to better interpret the mechanisms governing the transport, accumulation, and environmental fate of MPs in these coastal systems.

The concentration of MPs in Arad Bay, an ecotourism and protected area, were recorded as A1 (575 MP/kg d.w.), A2 (475 MP/kg d.w.), and A3 (550 MP/kg d.w.). The relatively moderate levels of MPs in this site may be attributed to regulated waste disposal and environmental management practices associated with its protected status. Land-based runoff via drainage outlets was identified as a potential source of MPs at this site, likely carrying MPs associated with urban surface wash-off, including tire wear particles, road dust, and plastic waste, consistent with the patterns documented in urban coastal environments.

While total MP abundance did not differ significantly between onshore and offshore sites (F ≈ 0, *p* = 0.987), an even stronger non-significant result than that observed between natural and afforested stands, a clear difference emerged in the particle size distribution and polymer composition along this same gradient. Onshore sites (Tubli Bay, Ras Sanad, and Arad Bay) exhibited MPs spanning the full detected size range (1.1–66.1 µm) and a comparatively diverse polymer assemblage, with PET detected across the entire size spectrum and Nylon-6 concentrated in the finer (<30 µm) fractions (Figure 3 and Figure 5). In contrast, the offshore Fasht Al-Jarim site was restricted to particles below 60 µm (2.0–57.4 µm) and dominated by a narrower polymer set (FEP, PTFE, and Nylon-6). This divergence suggests that, although total MP loading offshore is comparable to that recorded onshore, consistent with the tidal exchange and hydrodynamic connectivity already invoked to explain the ubiquity of Nylon-6 across typologies, the particles reaching Fasht Al-Jarim represent a size and composition-filtered subset (>60 µm) maybe preferentially retained onshore, where the dense root architecture and reduced flow velocities of vegetated mangrove sediments favor the deposition of coarser material close to the point of input, whereas finer, more buoyant or neutrally dense particles are more readily transported offshore before settling [63,64]. Similarly, the narrower polymer assemblage at Fasht Al-Jarim may reflect a combination of selective long-range transport, in which only certain polymer densities and degradation states persist over distance, and the relative absence of the diverse land-based inputs, sewage discharge, urban runoff, and mixed consumer waste streams that diversify the polymer pool at onshore sites. Taken together, these results indicated that onshore–offshore connectivity, rather than the presence or absence of local anthropogenic sources, primarily governs MP distribution in this system: total abundance is buffered by regional-scale hydrodynamic mixing, while particle size and polymer identity retain a signature of transport distance and source proximity.

Fragments were the dominant MP shape at every site (Figure 3), consistent with the prevalence of secondary MPs formed through the progressive weathering of larger plastic debris reported across marine and coastal studies [65,66,67,68] (Table S5). Within the Arabian Gulf, although studies investigating MPs in mangrove sediments remain relatively limited, available evidence consistently demonstrates the predominance of sub-millimeter particles. One of the earliest investigations by Naji et al. (2019) reported that MPs within the 10–300 µm size range constituted approximately 70–97% of the total particles detected in surface sediments from mangroves along the northern Arabian coasts [69]. More recently, a large-scale survey encompassing 24 sampling stations across eight mangrove ecosystems distributed along the Arabian Gulf and Gulf of Oman coastlines identified the 100–500 µm fraction as the most abundance size class [53]. Taken together, these studies indicate that particles smaller than 500 µm consistently dominate MP assemblages in Arabian Gulf mangrove sediments, likely reflecting the cumulative effects of prolonged environmental weathering, fragmentation, and extended residence times within these depositional environments. Similar size distributions have also been reported from mangrove ecosystems along the Red Sea and Arabian Gulf coasts of Saudi Arabia, where particles < 500 µm were likewise the predominant fraction, further emphasizing the capacity of mangrove habitats to function as efficient sinks for sub-millimeter MPs across diverse geographic settings [70]. By contrast, studies conducted in the United Arab Emirates, particularly within Abu Dhabi mangrove ecosystems, have predominantly reported MPs belonging to larger size classes (≥300 μm), a pattern that is more likely attributable to methodological detection limits than to the actual absence of smaller particles [71]. Most regional investigations have employed relatively coarse sieving thresholds (typically ≥300 μm), resulting in the systematic omissions of finer MP fractions from quantitative assessments. Against this backdrop, the predominance of MPs < 10 µm identified in the present study represents a previously overlooked and potentially major component of MP contamination in Arabian Gulf mangrove ecosystems. This observation challenges the conventional understanding of MP size distributions in the region and suggests that existing studies may have considerably underestimated both the abundance and ecological relevance of the smallest particles. The dominance of this ultra-fine fraction is indicative of extensive weathering and the fragmentation of plastic debris and reveals a substantial reservoir of particles characterized by high surface-area-to-volume ratios, enhanced contaminant adsorption potential, and increased bioavailability [72,73]. Owing to their minute size, these particles are also more readily internalized by biological tissues, including mangrove root systems, where they may interfere with physiological processes and compromise plant health [74,75]. The ecological significance of the ultrafine fraction extends beyond its abundance. Particles < 10 µm exhibit greater mobility within sediment pore water and an enhanced capacity to sorb co-occurring contaminants such as heavy metals and persistent organic pollutants, potentially acting as vectors that increase bioavailability and combined toxicological exposure for inhabitants [72,73,76]. Their minute size also accelerates uptake across root epidermal and iron-plaque barriers. Recent evidence in the mangrove species *Kandelia obovate* displays that nanoplastics can disturb root iron plaque formation and rhizophore microbial communities, processes probably relevant to the sub-10 µm particles described here [77]. These combined properties suggest that the ecological risk posed by this size fraction may be disproportionate to its mass, reinforcing the need for size-resolved risk assessment in mangrove sediments. Collectively, these findings underline an important methodological and conceptual limitation in current regional assessments and emphasize the need for standardized, high-resolution analytical techniques, capable of detecting the complete MP size spectrum in ecologically sensitive environments such as mangrove ecosystems.

Color is ecologically relevant, as well as diagnostic: particles resembling natural prey are more susceptible to ingestion, and color can indicate the origin and weathering history [78]. Black was the dominant color here, followed by brown, blue, red, grey, pink, and green, consistent with extensive weathering, organic and sediment fouling, and the common use and low recyclability of black plastic bags and packaging [79]. Brighter, less-weathered colors such as blue, red, and green more likely reflect fresher inputs from packaging, ropes, textiles, and fisheries gear, indicating multiple anthropogenic sources [66,80,81]. Progressive fading and darkening further reflect photo-oxidation, fouling, and sediment interactions typical of depositional mangrove settings [82,83,84].

Raman spectroscopy identified a diverse group of polymers, including Nylon-6, FEP, PTFE, PET, diamin-based compounds, MWCNT, PP, PU, and PS. This profile contrasts most Arabian Gulf studies, where MPs are typically dominated by PE and PP reflecting their widespread use and production [85,86]. The broader diversity observed in this study, particularly fluorinated polymers (FEP, PTFE) and MWCNT, suggests additional inputs from industrial and advance material sources that are rarely documented in the Gulf [87]. The presence of Nylon-6 is tied to fishing lines, nets, and ropes, as well as synthetic textiles; PP is tied to ropes, fishing gear, bottle caps, and packaging; PU aligns with foams and coatings; and PET/PS aligns with consumer packaging, beverage bottles, and disposable food service items.

The widespread occurrence of Nylon-6 at every sampling site, regardless of the site location (onshore or offshore) or mangrove type (natural or afforested), indicated the presence of a common and spatially extensive source rather than isolated point-source contamination [88]. In contrast to polymer types whose spatial distribution may be influenced by local environmental characteristics, such as the vegetation density, sediment grain size, or proximity to specific discharge outlets, Nylon-6 exhibited a remarkably consistent distribution across all environmental settings investigated [55]. Because Nylon-6 is strongly associated with fishing nets, ropes, fishing lines, and synthetic textile fibers, its ubiquitous presence suggests that these materials represent persistent and widely distributed sources of MP contamination throughout the study area. The regional dispersion of this polymer is likely enhanced by tidal exchange and hydrodynamic connectivity, which facilitate the transport and redistribution of particles between onshore and offshore mangrove habitats [89]. Furthermore, the detection of Nylon-6 within afforested mangrove sites, including Arad Bay and Fasht Al-Jarim, indicated that its occurrence is not solely controlled by local depositional conditions or habitat characteristics. Instead, this pattern supports the existence of regionally distributed external inputs that operate independently of site-specific environmental factors. Collectively, the broad spatial distribution of Nylon-6, together with its well-established association with fishing gear and synthetic textile fibers, identifies fishing-related activities and the environmental degradation of these materials as major and persistent contributors to MP contamination across Bahrain’s mangrove ecosystems, despite the environmental heterogeneity among sampling sites [66,67,88,89,90]. Additional to sources related to fishing, synthetic textile fibers can spread into coastal sediments through domestic and industrial wastewater, as Nylon-6-based garments release substantial quantities of microfibers during washing that are only partially retained by conventional treatment methods [91]. The closeness of the investigated mangrove sites to urban and semi-urban areas and treated or partially treated effluent discharge signifies a probable additional pathway supporting the abundant Nylon-6 signal observed here, alongside marine activities, boat maintenance, and other coastal servicing operations that similarly depend on nylon-based ropes and nets. Separating the relative contribution of these fishing-, textile-, and wastewater-derived pathways will require future source apportionment work exploring polymer fingerprinting with land-use and discharge-point mapping.

The observed decoupling between mangrove origin and pollution profiles is evident not only in the polymer composition but also in MP abundance. No significant difference in total MP abundance was observed between natural and afforested sites (F = 0.723, *p* = 0.415), despite the two afforested stands sampled here differing considerably in age: Arad Bay, afforested since 1998, was an approximately 27 years old at the time of sampling (2025), while Fasht Al-Jarim has no documented planting records, but is estimated to have been established around 2013, making it approximately 12 years old. This mirrors the Nylon-6 pattern described above: rather than reflecting a shortcoming in sampling design, it suggested that, even across this range of stand maturities, canopy closure, root mat development, and sediment accretion in the afforested stands have progressed sufficiently to approach those of naturally established stands. This raised a question worth stating explicitly: whether “natural” versus “afforested” still represents a functionally distinct ecological state in mangrove of this age, or whether it now serves primarily as a descriptor of stand origin and planting history rather than current ecosystem structure and function. A non-significant result does not, on its own establish ecological equivalence between stand types; however, the pattern at the older site is broadly consistent with the global synthesis of planted mangrove stands, showing biomass carbon stocks and structural attributed plateau at roughly 70–75% of intact natural-stand levels within 20 to 40 years of establishment, a trajectory that closely matches Arad Bay’s estimated age [92]. Fasht Al-Jarim, at an estimated 12 years, falls below this reported plateau window, yet showed no detectable difference in MP abundance either, suggesting that the convergence of MP abundance between afforested and natural stands may not depend solely on the structured maturity metrics captured by biomass-based syntheses. Taken together, this supports treating stand age as a relevant covariate in future MP assessments, one that may need to be considered independently for each afforested site, alongside, or instead of, a binary origin classification.

A global comparison of studies on mangrove sediments presented in Table S5 [53,62,69,70,93,94,95,96,97,98,99,100,101,102,103,104,105,106,107,108] indicates that PE and PP are the most frequently reported MP types, while fibers and fragments are the dominant shapes. In contrast, the present study identified Nylon-6, PET, FEP, and PTFE as the predominant polymer types, with fragments representing the dominant particle shape. This discrepancy may reflect two complementary factors. First, region-specific anthropogenic inputs, including industrial activities, fisheries, and urban waste streams characteristic of the Arabian Gulf, likely influence the polymer composition [81,89]. Second, methodological differences among studies probably relied on the visual identification or ATR-FTIR analysis of only a subset of particles, whereas the present study employed high-resolution Raman spectroscopy to characterize particles across the entire size range. The high spatial resolution and chemical sensitivity of Raman spectroscopy enable the reliable identification of smaller MPs and a broader range of polymer types, including fluoropolymers such as FEP and PTFE, which may remain undetected when lower resolution or size-restricted analytical techniques are employed [81,89,109]. Consequently, differences in reported polymer compositions among studies should be interpreted with caution, as the apparent absence of specific polymers may result from methodological constraints rather than their actual absence in the environment. Despite these analytical differences, studies from Iran and Pakistan have documented MP polymers, morphologies, and abundance patterns comparable to those observed in the present investigation, suggesting the influence of shared regional pollution sources and the close geographic connectivity of these coastal environments [53,108]. Overall, the polymer profile identified in this study appears to be more consistent with regional distribution patterns than with global trends, emphasizing the importance of standardized, high-resolution analytical methodologies to improve the reliability and comparability of future MP assessments across studies.

The occurrence of MPs in mangrove ecosystems presents an essential, environmental concern that affects the sediment structure, disrupts biological communities, and facilitates the accumulation and transfer of toxic contaminants, thus challenging essential mangrove ecosystem functions [8,110]. Consequently, these impacts may lead to a decline in the ecological health and resilience of mangrove habitats [95,111]. Additionally, MP contamination may adversely affect mangrove growth, seedling establishment, and long-term ecosystem sustainability through alterations in sediment quality and increased environmental stress on mangrove communities. Therefore, effective management strategies and restoration actions are required to support successful mangrove plantation programs in coastal areas.

In this study, the potential ecological risk of MP pollution in Bahrain mangrove ecosystems was evaluated by integrating PLI, PHI, and RI indices, which consider both MP abundance and polymer-specific toxicity. Overall, the risk was found to be at a low–moderate level (Figure 6). PLI values were consistent with MP abundance across the sampling sites. Although PHI values were in the moderate risk category at all sites, RI values remained low. This can be explained by the fact that the detected MPs mostly have low hazard scores, and hazard scores are not available for some MP types. This situation may create uncertainty in PHI- and RI-based assessments and may lead to an underestimation of the actual risk. A related limitation is that PLI, PHI, and RI are derived from polymer identity and particle abundance, without an explicit size-dependent toxicity term; as such, they may not adequately capture the biological effects associated with the ultra-fine (<10 µm) fraction that dominated this study, including enhanced cellular uptake, translocation, and adsorption-mediated co-toxicity [112]. The low–moderate risk classification reported here should therefore be interpreted as a conservative estimate rather than a definitive measure of ecological safety. Therefore, low MP abundance should not be directly interpreted as low ecological risk. In addition, the stronger relationship between RI and PLI (R^2^ = 0.60) compared to RI and PHI (R^2^ = 0.30) indicates that MP abundance has a greater influence on environmental risk than MP diversity in this study (Figure S4). This finding is consistent with results reported for mangrove ecosystems in the Arabian Gulf and the Gulf of Oman [53]. However, in sediment samples from Regional Organization for the Protection of The Marine Environment (ROPME) region, high PLI, PHI and PERI values have been reported, due to the presence of high-hazard MPs such as PVC, PA, and PS [113]. Therefore, the low–moderate risk observed in Bahrain may be explained by the limited presence of high-hazard MPs, and it shows that ecological risk depends not only on MP abundance but also strongly on MP composition. Nevertheless, the current risk level should not be interpreted as grounds for complacency, given the demonstrated uncertainties in hazard scoring and the potential for risk escalation with continued plastic inputs. Addressing this needed the application of thorough mitigation measures, incorporating robust environmental policies, permanent monitoring of MP pollution, and advanced waste management practices. Additionally, conservation initiatives intended at avoiding mangrove degradation by improving mangrove growth, regeneration, and seedling dispersal are crucial, as structurally and healthy intact mangrove ecosystems are well positioned to buffer against the accumulation of ecological pressures caused by MP contamination.

PCA (Supplementary Material, Figure S3) indicated that MP abundance, nitrate, and phosphate concentrations clustered along the same ordination axis, with Fasht Al-Jarim aligning closely with both nutrient variables and MP abundance. This co-occurrence is a statistical association only and should not be read as evidence that nutrient inputs drive MP accumulation. Elevated nitrate and phosphate at Fasht Al-Jarim more plausibly reflect agricultural runoff; whether agricultural activity also contributes MPs (e.g., mulch-film or irrigation-material fragments) remains speculative without dedicated source-apportionment data, and we flag it here only as a hypothesis for future work rather than a conclusion of this study. In contrast, ammonia was positioned in the opposite direction to MP abundance and other nutrients, indicating the influence of different sources or nitrogen transformation processes. Microbial communities on MP surfaces (plastisphere) can play a role in the nitrogen cycle or phosphorus release [110,111]. Therefore, the observed relationship with nitrates may be indirectly linked to microbial processes. However, since no microbial analyses were conducted in this study, this mechanism cannot be confirmed and should be considered only as a possible explanation. Further studies are needed to investigate MP–plastisphere interactions, particularly in this region.

### 4.2. Sustainability of Mangrove Ecosystems and Policy Relevance

Mangrove ecosystems reinforce several Sustainable Development Goals, SDG 13 (Climate Action), SDG 14 (Life Below Water), and SDG 15 (Life on Land), through carbon sequestration, biodiversity conservation, and coastal protection, alongside socioeconomic benefits from fisheries and other ecosystem services, and indirect contributions to SDG 3 through pollutant filtration and hazard mitigation [114,115,116,117,118,119]. Restoration programs in countries such as China, Bangladesh, and Indonesia illustrate how mangrove conservation advances these goals in practice, though weak policy integration, limited monitoring, and pressures from coastal development, aquaculture, and pollution continue to threaten long-term sustainability elsewhere [114,116,120,121,122].

Bahrain’s own restoration efforts, the Bahrain Mangroves Initiative and National Afforestation Strategy, which aim to quadruple mangrove coverage by 2035 as part of the Kingdom’s 2060 carbon-neutrality commitment, sit within this broader context of strengthened environmental governance and cross-sector partnership [123,124]. However, these sustainability gains cannot be fully realized while MP pollution accumulates unchecked: MP contamination has been shown to shift mangrove sediment microbial communities toward methanogenic pathways at the expense of blue-carbon storage [125], impair root oxygen and nutrient uptake and thus growth [126], and raise concerns for fisheries productivity and food security through trophic transfer [127]. Our detection of Nylon-6 and other polymers across both natural and afforested sites shows that afforestation alone does not confer protection from plastic contamination, underscoring the need to pair large-scale planting with explicit MP monitoring and source control.

We therefore recommend integrating MP monitoring, adapting internationally recognized protocols such as OSPAR’s Coordinated Environmental Monitoring Program and the GESAMP guidelines [81,128], directly into Bahrain’s mangrove initiatives, with clear institutional ownership, a defined sampling protocol, and measurable intervention thresholds, so that restoration outcomes translate into genuine ecological resilience rather than coverage alone.

Taken together, the consistent occurrence of MP contamination across both natural and afforested stands and across onshore and offshore settings indicates that the mangrove condition and shoreline exposure are poor predictors of contamination status in this system. This has a direct practical implication for the monitoring design: afforested plantations should not be treated as pollution-free reference sites, nor should offshore stands be assumed to be protected by distance from the coast. Because the hydrodynamic connectivity, fishing-dependent economies, and semi-enclosed basin structure driving Nylon-6 dispersal in Bahrain are shared across much of the Arabian Gulf, these findings offer a transferable basis for extending harmonized, ROPME-coordinated MP monitoring to mangrove systems throughout the region [53,88,129,130].

### 4.3. Limitations and Future Research

This baseline assessment has six main limitations. First, sampling covered four sites (12 stations) over a single 2025 campaign, limiting inferences about seasonal and interannual variability; broader, multi-year sampling is needed. Second, MP extraction used a saturated NaI solution (≈1.8 g cm^−3^), one of several floatation media (NaCl, NaBr, NaI, ZnCl_2_) that trade off recovery efficiency, cost, safety, and disposal, and differences among media likely contribute to cross-study discrepancies in reported abundance and composition; even with Raman spectroscopy, detection of the smallest and chemically complex particles remains uncertain, and complementary µ-FTIR or pyrolysis-GC/MS could improve confidence in this range. Third, cleaning water was not further filtered, a possible source of background contamination for the ultra-fine particles central to our findings; procedural blanks showed no overlap with Nylon-6, the dominant environmental polymer, but future work should filter water to 0.2–0.45 µm as standard practice. Fourth, sparse polymer-specific hazard data, especially for less-common polymers, likely underestimates the true ecological risk, and hazard databases need expansion. Fifth, the absence of standardized MP sampling, extraction, and analysis protocols continues to limit cross-study comparability and should be addressed through harmonized methods and rigorous QA/QC (procedural blanks, positive controls, inter-laboratory validation). Finally, the site descriptions provided in Section 2.1 (e.g., proximity to human activities and potential MP sources) reflect qualitative, field-based observations recorded during this baseline survey rather than systematically measured metrics; subsequent studies should incorporate quantitative geospatial and activity-based measures, such as GIS-derived distances to roads and other infrastructure, the local population density, and fishing-effort or vehicular-traffic counts, to support more rigorous, quantitative source attribution.

Beyond these methodological gaps, future work should extend beyond sediment to biological compartments, benthic invertebrates, fish, and avifauna, to evaluate trophic transfer, bioaccumulation, and ecological risk across coastal food webs, and apply cross-ecosystem modeling linking mangroves to tidal channels, the water column, and intertidal flats to better resolve MP transport, retention, and connectivity. Together, these priorities would move the field toward a more integrated, ecosystem-based framework for assessing MP contamination in coastal systems.

## 5. Conclusions

This study provides the first baseline assessment of MP contamination in Bahrain’s mangrove sediments, addressing a significant data gap for the Arabian Gulf. MPs were detected at all 12 stations across four sites (225–1900 particles kg^−1^ d.w.), confirming these sediments as a persistent plastic sink; fragments were the dominant shape and black the dominant color, reflecting the extensive weathering of larger debris. The predominance of the <10 µm and 10–30 µm fractions, including a sub-10 µm class not previously documented in Gulf mangroves, indicates that conventional, coarser-threshold monitoring has likely underestimated both MP abundance and the ecological risk posed by ultra-fine particles, which combine high surface reactivity, enhanced adsorption capacity, and greater potential for root uptake and trophic transfer. Nylon-6 predominated at every site regardless of onshore or offshore setting, pointing to common, persistent anthropogenic sources. Ecological risk indices (PLI, PHI, RI) classified overall risk as low-to-moderate, but because these indices do not account for particle size, this classification likely underestimates the true risk posed by the ultra-fine fraction. These findings establish a robust baseline for future spatiotemporal monitoring and underscore the need for standardized, high-resolution detection methods; priority research directions include the bioaccumulation and trophic transfer of ultra-fine MPs, their long-term ecological and toxicological impacts, and the identification of dominant sources and transport pathways, providing the evidence base for the targeted management of mangrove and other coastal ecosystems across the Arabian Gulf.

## Figures and Tables

**Table 1:** Sites and stations’ information in the sampling mangroves areas.

Sampling Site	Station	Latitude	Longitude	Natural or Afforested, Onshore or Offshore	Key Value of Mangrove Site
Arad Bay	A1	26.263743	50.624409	Afforested– onshore	National Mangrove expansion; ecotourism park Protected Area
	A2	26.262815	50.629133		
	A3	26.261429	50.632489		
Tubli Bay	T1	26.153119	50.607417	Natural—Onshore	Ramsar site; Protected Area
	T2	26.154947	50.607994		
	T3	26.153849	50.606617		
Ras Sanad	R1	26.153674	50.594176	Natural—Onshore	Ramsar site
	R2	26.154922	50.593525		
	R3	26.147434	50.591809		
Fasht Al-Jarim	J1	26.422348	50.494746	Afforested— offshore	Under ecological investigation
	J2	26.420640	50.494057		
	J3	26.419560	50.496493		

**Table 2:** Grain size sediments collected at different stations in the selected mangrove sites.

Mangrove Site	Mangrove Station	Grain Size (%)				Dominant Of Sediment Type
		Pebble	Sand	Silt	Clay	
Tubli Bay	T1	0.17	70.93	10.5	18.40	Sand
	T2	4.20	84.31	7.29	4.20	Sand
	T3	0.43	89.47	5.80	4.30	Sand
Ras Sanad	R1	2.60	79.20	14.10	4.10	Sand
	R2	11.59	72.55	8.02	7.84	Sand
	R3	1.00	78.30	16.84	3.86	Sand
Arad Bay	A1	1.91	67.50	20.59	10.00	Sand
	A2	2.20	74.14	9.82	13.84	Sand
	A3	4.15	66.19	24.06	5.60	Sand
Fasht Al-Jarim	J1	6.48	73.82	16.14	3.56	Sand
	J2	1.43	63.21	31.79	3.57	Sand
	J3	17.6	79.64	2.03	0.73	Sand

## Data Availability

The original contributions presented in this study are included in the article/Supplementary Material. Further inquiries can be directed to the corresponding authors.

## Supplementary Materials

The following supporting information can be downloaded at https://www.mdpi.com/article/10.3390/toxics14090833/s1, Figure S1: Raman spectra of identified particles. The assigned MPs are (a) copper phthalocyanine-PP, (b) FEP, (c) Nylon-6, (d) PET, (e) PET/diamine/MWCNT composite, (f) PP, (g) PS, (h) PTFE, (i) PU, (j) PU/PP blend. (k) A particle-free area was scanned under Raman microscope to be used as a blank spectrum.; Table S1: Fingerprint bands for assigned MPs; Figure S2. Raman spectrum of the PS positive control particle obtained from the PS reference sample filtered onto a GF/F filter; Table S2: Microplastic abundance (MP/kg) and associated physicochemical water quality parameters measured at the sampling locations; Figure S3. PCA analysis of MP abundance and physicochemical parameters across study sites in the Kingdom of Bahrain; Table S3: Classification criteria for PLI, PHI, and RI index; Table S4: Results of different risk indices in the sampling stations; Figure S4: Scatterplot of RI, PLI, and PHI; Table S5: Global comparison of Microplastics in mangrove sediments. References [37,38,39,40,41,42,43,44,45,46,47,48,49,50,51,53,69,70,93,94,95,96,97,98,99,100,101,102,103,104,105,106,107,108,130] cited in supplementary materials.
