# Supplementary material for: Microplastics Contamination and Ecological Risk Assessment in Natural and Afforested Mangrove Stands of Bahrain: Abundance, Polymer Composition, and Onshore–Offshore Variability
Source: Toxics. 2026 Sep 20;14(9):833. doi: 10.3390/toxics14090833 (PMC13611499; doi:10.3390/toxics14090833)
Supplement: Supplementary file 1 [file toxics-14-00833-s001.zip › toxics-4509742-supplementary.pdf]

Supplementary Materials

Microplastics Contamination and Ecological Risk Assessment in Natural and Afforested Mangrove Stands of Bahrain: Abundance, Polymer Composition, and Onshore–Offshore Variability

Zeynep Kilinc[1,2], Gamze Yesilay*[1,2], Abdulkarim Rashed[3], Reem AlMealla[4], Batool Ahmed[5], Sakina Mustafa[5], Ahmed Khamis[6], Khalil Abdulla [6], Layla Hazeem*[5]

[1] Department of Molecular Biology and Genetics, University of Health Sciences-Turkey, Istanbul 34668, Türkiye
[2] Experimental Medicine Application & Research Center, University of Health Sciences-Turkey, Validebag Research Park, Istanbul 34662, Türkiye
[3] Independent Researcher in Environment and Sustainable Development, Manama 18165, Bahrain
[4] Nuwat for Environmental Research & Education, Al Janabiyah, Kingdom of Bahrain
[5] Department of Biology, College of Science, University of Bahrain, PO Box 32038, Sakhir, Kingdom of Bahrain
[6] Supreme Council for the Environment (SCE), PO Box 18233, Manama, Kingdom of Bahrain

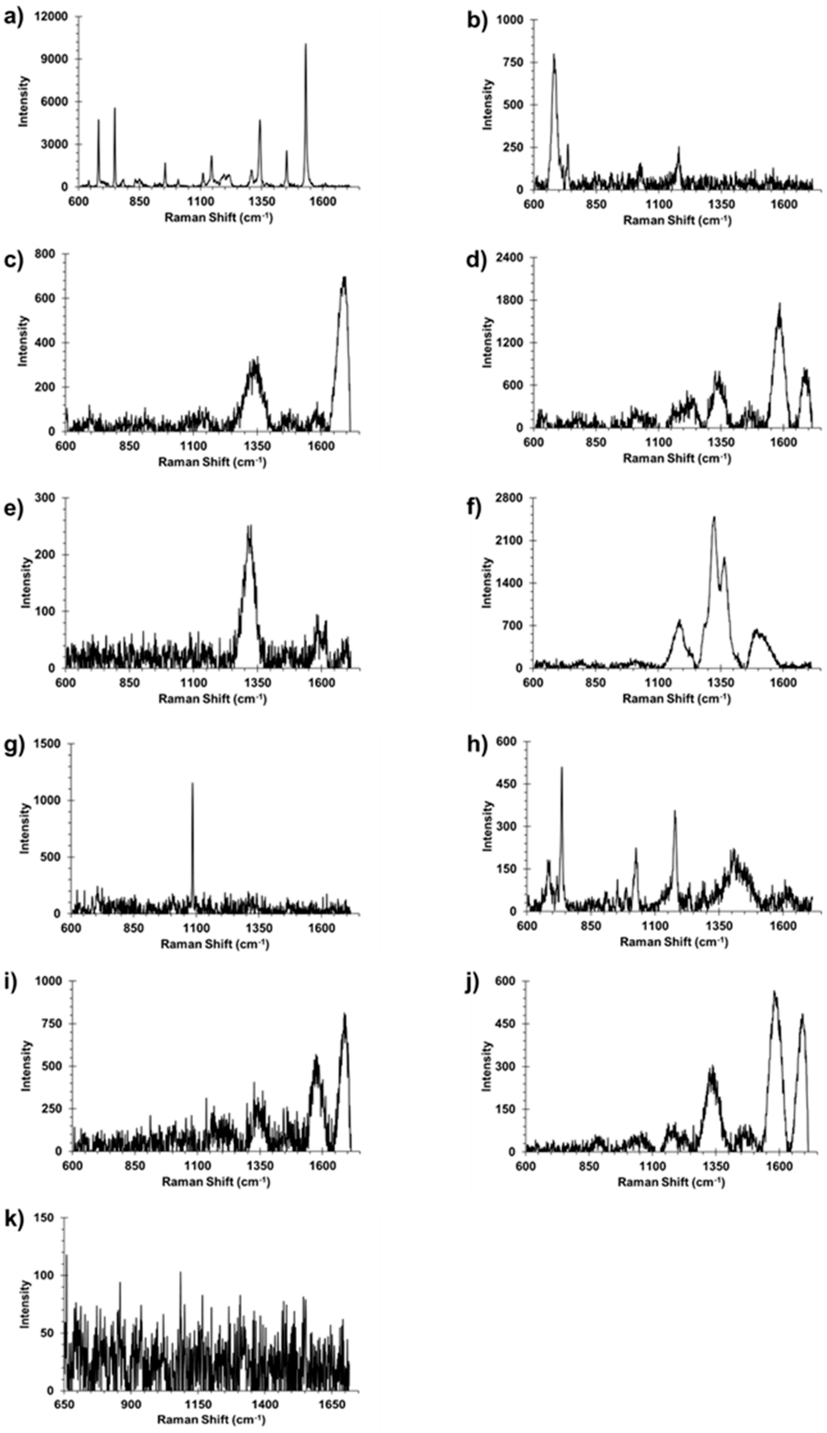

Figure S1. Raman spectra of identified particles. The assigned MPs are **(a)** copper phthalocyanine-PP, **(b)** FEP, **(c)** Nylon-6, **(d)** PET, **(e)** PET/diamine/MWCNT composite, **(f)** PP, **(g)** PS, **(h)** PTFE, **(i)** PU, and **(j)** PU/PP blend. **(k)** A particle-free area was scanned under Raman microscope to be used as a blank spectrum.

Table S1. Fingerprint bands for assigned MPs.

MP Type	Wavenumber (cm^{-1})	Peak Assignment	Ref.
Copper phthalocyanine-PP	839	Rocking of CH_2 and CH_3 moieties, stretching of CC and C-CH_3 moieties	[1]
	954	Rocking of CH_3 moieties	
	1451	Bending of CH_3 and CH_2 moieties	
	681, 1145, 1342, 1530	Copper phthalocyanine	
FEP	680	CF_3 bond vibration	[2,3]
Nylon-6	1696	Amide I, C=O stretch	[4,5]
	1475	Amide II, C-N Stretch, N-H Bend	
	1334	Amide III, C-N Stretch, N-H Bend, CH_2 Twist	
	1125	C-C Skeletal Stretch	
	911	C-CO Stretch	
PET	1691	C=O bond	[6]
	1585	C-C bond	
PET/diamine/ MWCNT	1323	D band, defects in carbon structure	[7]
	1590	G band	
PP	1187	C–C stretching, CH_3 bending	[8–10]
	1325	CH stretching, CH_2 wagging, CH_3 bending	
	1498	CH_2 bending, CH_3 asymmetric bending	
PS	1084	Aromatic ring	[11]
	1125	C-H in-plane deformation	
	1509	Skeletal stretching mode	
PTFE	736	CF_2 stretching	[12,13]
PU	1687	Vibration of amide II group	[14]
PU/PP Blend	1462	CH_3 asymmetric bending	[15]
	1688	Vibration of amide II group	

NB: Copper phthalocyanine-polypropylene (copper phthalocyanine-PP), fluorinated ethylene propylene (FEP), poly (ethylene terephthalate) (PET), PET/diamine/multi-walled carbon nanotube (MWCNT) composites (PET/diamine/MWCNT), polystyrene (PS), polytetrafluoroethylene (PTFE), polyurethane (PU).

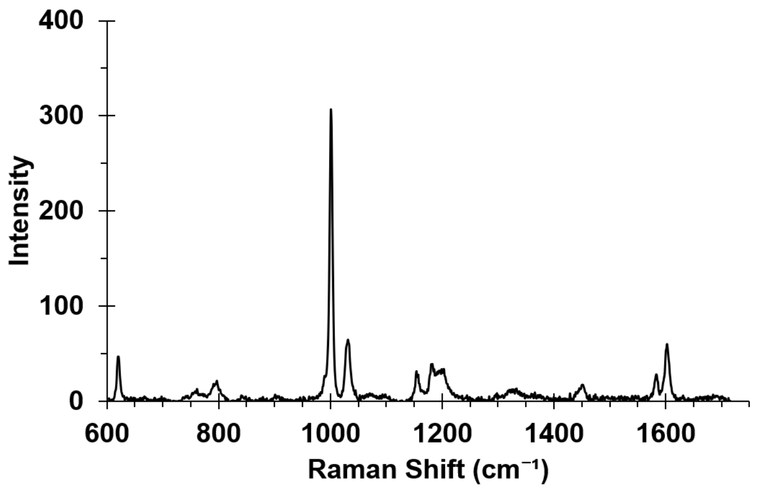

Figure S2. Raman spectrum of the PS positive control particle obtained from the PS reference sample filtered onto a GF/F filter.

Table S2. Microplastic abundance (MP/kg) and associated physicochemical water quality parameters measured at the sampling locations.

Location	Code	Abundance (MP/kg)	Ammonia (mg/L)	Phosphate (mg/L)	Nitrate (mg/L)	Temperature (°C)	DO (%)	Salinity (PSU)	pH
Tubli Bay	T1	650	0,49	0,32	0,19	15,90	86,50	45,66	7,93
	T2	550	0,50	0,14	0,23	16,70	81,50	58,51	7,75
	T3	875	0,50	0,45	0,23	19,20	119,90	44,60	8,48
Ras Sanad	R1	800	0,52	0,69	0,35	16,50	139,70	53,91	8,19
	R2	1125	0,51	0,20	0,38	15,80	51,10	46,87	7,93
	R3	950	0,29	0,11	0,25	18,80	93,90	43,30	8,23
Arad	A1	575	0,41	0,23	0,01	17,20	125,10	45,18	8,34
	A2	475	0,47	0,32	0,01	18,50	131,10	46,73	8,52
	A3	550	0,73	0,26	0,01	24,60	131,90	39,54	8,03
Al Jarim	J1	225	0,47	0,31	0,21	34,80	159,80	44,04	8,40
	J2	1900	0,40	0,35	0,26	35,90	143,30	46,64	8,40
	J3	475	0,43	0,32	0,29	31,90	126,80	43,53	8,38

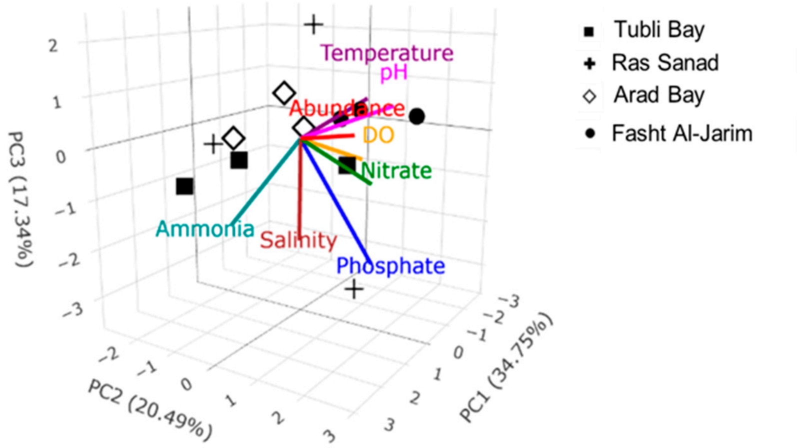

Figure S3. PCA analysis of MP abundances and physicochemical parameters across study sites in the Kingdom of Bahrain.

Table S3. Classification criteria for the PLI, PHI, and RI index.

**Risk Index**	**Risk Categories**			
	**I (Low)**	**II (Medium)**	**III (High)**	**IV (Extremely High)**
PLI	<1	1-2	2-3	>3
PHI	<10	10-100	100-1000	>1000
RI	<150	150-300	300-3000	>3000

Note: the risk categories is taken from Kor et al. 2026. [16]

Table S4. Results of different risk indices in the sampling stations.

**Mangrove site**	**Station**	**Abundance (particle.kg^{-1})**	**PLI**	**PHI**	**RI**
Tubli Bay	T1	650	1,20	24,44	29,42
	T2	550	1,02	43,43	44,23
	T3	875	1,62	27,84	45,11
Ras Sanad	R1	800	1,48	23,61	34,98
	R2	1125	2,08	32,74	68,21
	R3	950	1,76	39,42	69,34
Arad Bay	A1	575	1,06	43,04	45,83
	A2	475	0,88	35,32	31,07
	A3	550	1,02	32,49	33,09
Fasht Al-Jarim	J1	225	0,42	4,00	1,67
	J2	1900	3,52	41,71	146,74
	J3	475	0,88	43,87	38,59

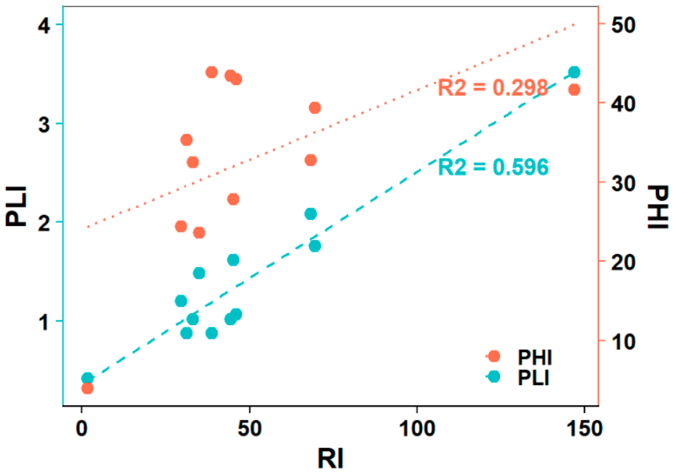

Figure S4. Scatterplot of RI, PLI, and PHI.

Table S5: Global comparison of microplastics in mangrove sediments.

Country	Visual inspection	Instrumental analysis	Density (particles. Kg-1) Min-Max	Major types	Major components	Ref.
Brazil	Stereoscope	Systematic photographic mapping and digital processing	555- 31,087	Fibers, Fragments	PE, PP, PS, PET, and PVC	[17]
Brazil	Stereomicroscope	Micro-Raman and FTIR spectroscopy	6,150 – 93,050	Fibers, Lines, Fragments, Foams	PE, PP, PS, PET, PU, PMMA, and PA	[18]
China	Stereomicroscope	FT-IR, μ-FTIR, SEM-EDS	8.3- 5,738.3	Foams, Fibers	PS, PP	[19]
China	Raman microscope	ATR-FT-IR, Raman spectroscopy	47- 35,000	Foams, Fibers	PS, PP, PE	[20]
China	Stereomicroscope	FT-IR, SEM-EDS	0- 3,123.3	Fiber	PP, PE	[21]
China	Stereomicroscope	FTIR, LDIR	374.3 – 7,475.3	Synthetic rubbers, Fibers, and non-Fibers	PP, PE, PET, FMQ (Fluorosilicone rubber), and PU	[22]
Colombia	Stereoscope	-	42 – 1,825	Films, Foams, Fragments	Not specified	[23]
India	Stereomicroscope	Confocal Raman Microscope	1,275 ± 532 (sediment average)	Fragments, Fibers	LDPE, PP, Polypropylene	[24]

India	Stereomicroscope	FTIR-ATR	9.33 – 27	Fibers, Fragments	PE, PP	[25]
India	Binocular digital microscope	Raman-AFM Microscope, SEM-EDS	235 – 1,414	Fragments, Fibers	PE, PP	[26]
Indonesia	Stereomicroscope	ATR-FTIR	11.83 – 47.79	Foams, Fragments	PS, PP, PE	[27]
Indonesia	Microscope	ATR-FTIR	9.62 – 103.17	Fragments, Fibers	LDPE, PP, PS	[27]
Indonesia	Microscope	-	1,140 – 3,840	Pellets, Fibers	Not explored	[28]
Iran	Stereomicroscope	FE-SEM - EDX, FTIR	19.5 - 34.5	Microfibres, fragments	PE	[29]
Iran	Optical microscope	Micro-Raman spectrometer	38 – 1280	Fibers, Fragments	PE, PP, Polyamide	[16]
Iraq	Stereomicroscope	FTIR	500 – 1300	Fragments, Fibers	PE, PP	[30]
Malaysia	Stereomicroscope	ATR-FTIR, FTIR Microscope	63 – 140	Fragments, Films, Fibers	Rayon, PE	[31]
Malaysia	Stereomicroscope	ATR-FTIR, SEM-EDS	185 – 800	Fibers, Fragments	PP	[32]
Oman	Stereomicroscope	ATR-FTIR	6 – 256	Fragments, Pellets, Fibers	PE, HDPE	[33]
Pakistan	Stereomicroscope	FTIR	830 (mean)	Beads, Films, Fibers, Fragments	PET, PE	[34]
Saudi Arabia	Dissecting microscope	FTIR and μFTIR	84 (mean)	Fragments, Films, Threads	PE, PP	[35]

References

1. Ragusa, A.; Svelato, A.; Santacroce, C.; Catalano, P.; Notarstefano, V.; Carnevali, O.; Papa, F.; Rongioletti, M.C.A.; Baiocco, F.; Draghi, S.; et al. Plasticenta: First Evidence of Microplastics in Human Placenta. *Environ. Int.* **2021**, *146*, 106274. https://doi.org/10.1016/j.envint.2020.106274.
2. Fujitani, K.; Takeuchi, M.; Haruyama, Y.; Yamaguchi, A.; Utsumi, Y. Anisotropic Pyrochemical Dry Etching of Fluorinated Ethylene Propylene Induced by Pre-Irradiation with Synchrotron Radiation. *AIP Adv.* **2021**, *11*, 025104. https://doi.org/10.1063/6.0000702.
3. Takeuchi, M.; Kinoshita, A.; Yamaguchi, A.; Utsumi, Y. Molecular Structure Evaluation of Bulk Polytetrafluoroethylene Modified by X-Ray Irradiation. *J. Photopolym. Sci. Technol.* **2020**, *33*, 295–299. https://doi.org/10.2494/photopolymer.33.295.
4. Yuan, D.; Corvianawatie, C.; Cordova, M.R.; Surinati, D.; Li, Y.; Wang, Z.; Li, X.; Li, R.; Wang, J.; He, L.; et al. Microplastics in the Tropical Northwestern Pacific Ocean and the Indonesian Seas. *J. Sea Res.* **2023**, *194*, 102406. https://doi.org/10.1016/j.seares.2023.102406.
5. Miller, J.V.; Bartick, E.G. Forensic Analysis of Single Fibers by Raman Spectroscopy. *Appl. Spectrosc.* **2001**, *55*, 1729–1732. https://doi.org/10.1366/0003702011954099.
6. Polymer Analysis Using Raman Spectroscopy. *PhysicsOpenLab*.
7. Jin, S.H.; Park, Y.-B.; Yoon, K.H. Rheological and Mechanical Properties of Surface Modified Multi-Walled Carbon Nanotube-Filled PET Composite. *Compos. Sci. Technol. 2007, 67, 3434–3441. https://doi.org/10.1016/j.compscitech.2007.03.013.*
8. Lenz, R.; Enders, K.; Stedmon, C.A.; Mackenzie, D.M.A.; Nielsen, T.G. A Critical Assessment of Visual Identification of Marine Microplastic Using Raman Spectroscopy for Analysis Improvement. *Mar. Pollut. Bull.* **2015**, *100*, 82–91. https://doi.org/10.1016/j.marpolbul.2015.09.026.
9. Vasudeva, M.; Adarsh, U.K.; Warrier, A.K.; George, S.D.; Unnikrishnan, V.K. Performance Evaluation of a Hyphenated Laser Spectroscopy System with Conventional Methods for Microplastic Analysis. *Sci. Rep.* **2024**, *14*, 19327. https://doi.org/10.1038/s41598-024-70501-8.
10. Umurhan, Y.; Songsart-Power, M.; Limbu, T.B.; Phan, T. Applications of Raman Spectroscopy for Microplastic Detection and Characterization: A Comprehensive Spectral Reference. *Environ. Sci. Pollut. Res.* **2025**, *32*, 28630–28677. https://doi.org/10.1007/s11356-025-37224-3.
11. Nava, V.; Frezzotti, M.L.; Leoni, B. Raman Spectroscopy for the Analysis of Microplastics in Aquatic Systems. *Appl. Spectrosc.* **2021**, *75*, 1341–1357. https://doi.org/10.1177/00037028211043119.
12. Lux, A.; Conti, C.; Botteon, A.; Mosca, S.; Matousek, P. Shifted-Excitation Raman Difference Spectroscopy and Charge-Shifting Detection Coupled with Spatially Offset Raman Spectroscopy for Heritage Science. *Analyst* **2025**, *150*, 1140–1150. https://doi.org/10.1039/D4AN01280A.
13. Liu, K.; Pang, X.; Chen, H.; Jiang, L. Visual Detection of Microplastics Using Raman Spectroscopic Imaging. *Analyst* **2024**, *149*, 161–168. https://doi.org/10.1039/D3AN01270K.
14. Chakraborty, I.; Banik, S.; Biswas, R.; Yamamoto, T.; Noothalapati, H.; Mazumder, N. Raman Spectroscopy for Microplastic Detection in Water Sources: A Systematic Review. *Int. J. Environ. Sci. Technol.* **2022**, *20*, 10435–10448. https://doi.org/10.1007/s13762-022-04505-0.
15. Vinay Kumar, B.N.; Löschel, L.A.; Imhof, H.K.; Löder, M.G.J.; Laforsch, C. Analysis of Microplastics of a Broad Size Range in Commercially Important Mussels by Combining FTIR and Raman Spectroscopy Approaches. *Environ. Pollut.* **2021**, *269*, 116147. https://doi.org/10.1016/j.envpol.2020.116147.
16. Kor, K.; Ghaemi, M.; Ershadifar, H.; Koochaknejad, E.; Ghazilou, A.; Saleh, A.; Hamzeh, M.A. Comprehensive Assessment of Microplastic Contamination and Ecological Risks in Mangrove Sediments along the Coasts of the Persian Gulf and Gulf of Oman. *Mar. Pollut. Bull.* **2026**, *224*, 119165. https://doi.org/10.1016/j.marpolbul.2025.119165.
17. Paes, E. da S.; Gloaguen, T.V.; Silva, H. dos A. da C.; Duarte, T.S.; de Almeida, M. da C.; Costa, O.D.V.; Bomfim, M.R.; Santos, J.A.G. Widespread Microplastic Pollution in Mangrove Soils of Todos Os Santos Bay, Northern Brazil. *Environ. Res.* **2022**, *210*, 112952. https://doi.org/10.1016/j.envres.2022.112952.
18. Parra, D.F.; Gimiliani, G.T.; dos Santos, J.L.; Wetter, N.U.; Schepis, W.R.; Bereczki, A.; Cotrim, M.E.B. Microplastics in Santos São Vicente Estuarine—Hotspot in Sediments Caused by Low Energy Hydrodynamic Events in Strongly Populated Areas. *Mar. Pollut. Bull.* **2025**, *210*, 117286. https://doi.org/10.1016/j.marpolbul.2024.117286.
19. Zhou, Q.; Tu, C.; Fu, C.; Li, Y.; Zhang, H.; Xiong, K.; Zhao, X.; Li, L.; Waniek, J.J.; Luo, Y. Characteristics and Distribution of Microplastics in the Coastal Mangrove Sediments of China. *Sci. Total Environ.* **2020**, *703*, 134807. https://doi.org/10.1016/j.scitotenv.2019.134807.
20. Dou, P.-C.; Mai, L.; Bao, L.-J.; Zeng, E.Y. Microplastics on Beaches and Mangrove Sediments along the Coast of South China. *Mar. Pollut. Bull.* **2021**, *172*, 112806. https://doi.org/10.1016/j.marpolbul.2021.112806.

21. Yu, L.; Li, R.; Chai, M.; Li, B. Vertical Distribution, Accumulation, and Characteristics of Microplastics in Mangrove Sediment in China. *Sci. Total Environ.* **2023**, *856*, 159256. https://doi.org/10.1016/j.scitotenv.2022.159256.
22. Qiao, K.; Wang, W.-X. The Dual Role of Coastal Mangroves: Sinks and Sources of Microplastics in Rapidly Urbanizing Areas. *J. Hazard. Mater.* **2024**, *480*, 136408. https://doi.org/10.1016/j.jhazmat.2024.136408.
23. Garcés, O. Microplastic Pollution in Mangroves and Beaches of the Cispata Marine Protected Area, Colombian Caribbean Coast. *Rev. Cienc. Mar. Costeras* **2022**, *14*, 9–25.
24. Palat Kannankai, M.; Alex, R.; Muralidharan, V.; Nazeerkhan, N.; Radhakrishnan, A.; Devipriya, S. Urban Mangrove Ecosystems Are under Severe Threat from Microplastic Pollution: A Case Study from Mangalavanam, Kerala, India. *Environ. Sci. Pollut. Res.* **2022**, *29*, 80568–80580. https://doi.org/10.1007/s11356-022-21530-1.
25. Shelciya, S.; Glen Esmeralda, V.; Patterson, J. Preliminary Study on the Role of Mangroves in Entrapping Microplastics in Tuticorin Coast of Gulf of Mannar, Southeast Coast of India. *Arch. Environ. Contam. Toxicol.* **2023**, *85*, 25–33. https://doi.org/10.1007/s00244-023-01007-z.
26. Roshni, K.; Renjithkumar, C.R.; Amal, R.; Devipriya, S.P. Characterization and Risk Assessment of Microplastics Accumulated in Sediments and Benthic Molluscs in the Mangrove Wetlands along the South-West Coast of India. *Mar. Pollut. Bull.* **2025**, *216*, 117955. https://doi.org/10.1016/j.marpolbul.2025.117955.
27. Cordova, M.R.; Ulumuddin, Y.I.; Lubis, A.A.; Kaisupy, M.T.; Wibowo, S.P.A.; Subandi, R.; Yogaswara, D.; Purbonegoro, T.; Renyaan, J.; Nurdiansah, D.; et al. Microplastics Leaving a Trace in Mangrove Sediments Ever since They Were First Manufactured: A Study from Indonesia Mangroves. *Mar. Pollut. Bull.* **2023**, *195*, 115517. https://doi.org/10.1016/j.marpolbul.2023.115517.
28. Siregar, L.Y.; Yucharoen, M.; Muchlisin, Z.A.; Pradit, S. ACCUMULATION OF MICROPLASTICS (<300 μM) IN MANGROVE SEDIMENTS OF BANDA ACEH CITY, INDONESIA. *Appl. Ecol. Environ. Res.* **2025**, *23*, 3245–3258. https://doi.org/10.15666/aeer/2302_32453258.
29. Naji, A.; Nuri, M.; Amiri, P.; Niyogi, S. Small Microplastic Particles (S-MPPs) in Sediments of Mangrove Ecosystem on the Northern Coast of the Persian Gulf. *Mar. Pollut. Bull.* **2019**, *146*, 305–311. https://doi.org/10.1016/j.marpolbul.2019.06.033.
30. Mohammed, H.K.; Ghazi, A.H.; Al-Saad, H.T. Assessment of Microplastic Pollution in Sediments from Khor Al-Zubair, North-West of Arabian Gulf. Egypt. J. Aquat. Biol. Fish. 2025, 29, 3355–3373. https://doi.org/10.21608/ejabf.2025.436276.
31. Mohan, P.; Shahul Hamid, F. Charting the Microplastic Menace: A Bibliometric Analysis of Pollution in Malaysian Mangroves and Polypropylene Bioaccumulation Assessment in Anadara Granosa. *Mar. Pollut. Bull.* **2024**, *205*, 116654. https://doi.org/10.1016/j.marpolbul.2024.116654.
32. Maulana, N.; Zulkifli, M.; Khalid, A.; Rohani, S.; Anuar, S.; Jaafar, M.; Ariffin, E. Revealing Microplastic Contamination in Mangrove Sediments from Setiu Wetlands, Malaysia. *Indones. J. Chem.* **2025**, *25*, 520. https://doi.org/10.22146/ijc.100813.
33. Al-Tarshi, M.; Dobretsov, S.; Gallardo, W. Marine Litter and Microplastic Pollution in Mangrove Sediments in the Sea of Oman. *Mar. Pollut. Bull.* **2024**, *201*, 116132. https://doi.org/10.1016/j.marpolbul.2024.116132.
34. Riaz, S.; Sahar, R.; Qader, I.; Burhan, Z.-N.; Alvi, S.K.; Rasool, S.G.; Siddiqui, P.J.A.; Shafique, S. Preliminary Assessment of Microplastic in Rhizosphere and Non-Rhizosphere Region of Mangrove at Four Locations along Karachi Coast, Pakistan. *Mar. Pollut. Bull.* **2024**, *205*, 116608. https://doi.org/10.1016/j.marpolbul.2024.116608.
35. Martin, C.; Baalkhuyur, F.; Valluzzi, L.; Saderne, V.; Cusack, M.; Almahasheer, H.; Krishnakumar, P.K.; Rabaoui, L.; Qurban, M.A.; Arias-Ortiz, A.; et al. Exponential Increase of Plastic Burial in Mangrove Sediments as a Major Plastic Sink. *Sci. Adv.* **2020**, *6*, eaaz5593. https://doi.org/10.1126/sciadv.aaz5593.
